# Transcription factor networks disproportionately enrich for heritability of blood cell phenotypes

**DOI:** 10.1126/science.ads7951

**Published:** 2025-04-03

**Authors:** Jorge Diego Martin-Rufino, Alexis Caulier, Seayoung Lee, Nicole Castano, Emily King, Samantha Joubran, Marcus Jones, Seth R. Goldman, Uma P. Arora, Lara Wahlster, Eric S. Lander, Vijay G. Sankaran

**Affiliations:** 1. Division of Hematology/Oncology, Boston Children’s Hospital and Department of Pediatric Oncology, Dana-Farber Cancer Institute, Harvard Medical School, Boston, MA, USA.; 2. Howard Hughes Medical Institute, Boston, MA, USA.; 3. Broad Institute of MIT and Harvard, Boston, MA, USA.; 4. Nascent Transcriptomics Core, Department of Biological Chemistry and Molecular Pharmacology, Harvard Medical School, Boston, MA, USA.; 5. Department of Biology, Massachusetts Institute of Technology, Cambridge, MA, USA.; 6. Department of Systems Biology, Harvard Medical School, Boston, MA, USA.; 7. Harvard Stem Cell Institute, Cambridge, MA, USA.

## Abstract

Most phenotype-associated genetic variants map to non-coding regulatory regions of the human genome, but their mechanisms remain elusive in most cases. We developed a highly efficient strategy, Perturb-multiome, to simultaneously profile chromatin accessibility and gene expression in single cells with CRISPR-mediated perturbation of master transcription factors (TFs). We examined the connection between TFs, accessible regions, and gene expression across the genome throughout hematopoietic differentiation. We discovered that variants within TF-sensitive accessible chromatin regions in erythroid differentiation, while representing <0.3% of the genome, show a ~100-fold enrichment for blood cell phenotype heritability; substantially higher than for other accessible chromatin regions. Our approach facilitates large-scale mechanistic understanding of phenotype-associated genetic variants by connecting key *cis*-regulatory elements and their target genes within gene regulatory networks.

Human genetic studies have uncovered tens of thousands of genetic variants associated with complex diseases and phenotypes. With increasingly large sample sizes, the fraction of heritability explained is approaching saturation (1–4). However, genetic mapping fails to identify the mechanisms by which variants impact phenotypes, limiting biological understanding and the development of therapeutic strategies (5).

Base editing can assess the effect of variants and connect them with target genes and molecular pathways implicated in human disease. While there has been recent progress in achieving high-throughput base editing (6–10), systematic analysis of all variants of interest is not currently feasible.

Another approach leverages the fact that most trait-associated variants are located in non-coding regulatory elements of the genome, where they alter transcription factor (TF) and related protein binding and thereby regulate gene expression (11–13). A powerful way to study the mechanisms influenced by genetic variation could thus be to modulate TFs and simultaneously read out the genome-wide consequences on chromatin accessibility of *cis*-regulatory elements and gene expression (fig. 1A). To assess many TFs across diverse cell states involved in physiology and disease (14, 15), such studies should be performed at single-cell resolution and in disease-relevant primary cells (16, 17).

Here, we developed Perturb-multiome to achieve this goal and apply it to study perturbations of key TFs in primary human hematopoietic stem and progenitor cells (HSPCs), which can then be differentiated to produce mature red and other blood cells. We find that the regulatory regions in erythroid differentiation whose chromatin accessibility is sensitive to perturbation of these TFs (“TF-sensitive regulatory regions”) comprise <0.3% of all regions in the genome, but are enriched by nearly 100-fold for SNPs associated with certain blood-cell phenotypes. Our framework can be applied to human diseases and phenotypes beyond hematopoiesis to advance the understanding of genetic variants and the mechanisms underlying inherited risk for complex diseases.

## Results

### Pooled transcription factor perturbation screens to uncover coordinated chromatin accessibility and RNA alterations.

**Figure 1.**
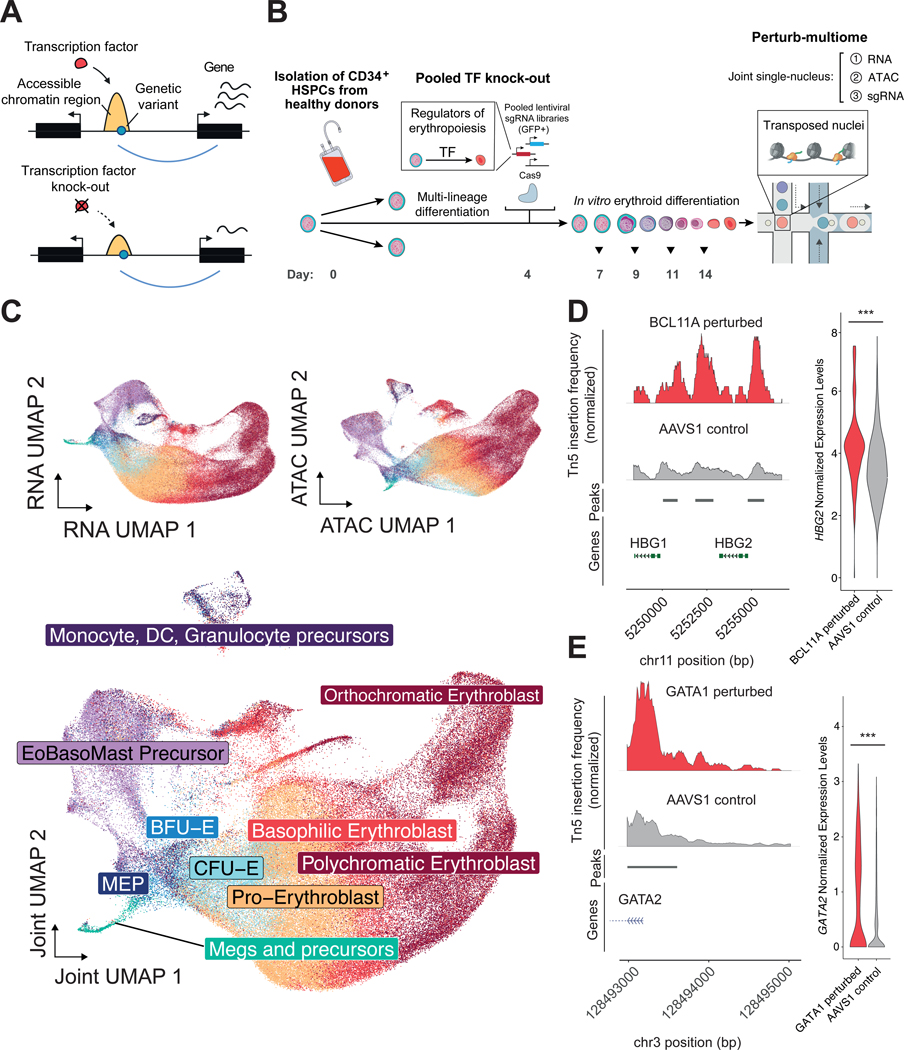
Perturb-multiome: pooled CRISPR screens with coupled single-cell RNA and chromatin accessibility readouts to profile primary human hematopoiesis. (A) Top: simplified representation of a basic gene regulatory network in which a transcription factor (TF) modulates chromatin accessibility of a *cis*-regulatory element (enriched in trait- or disease-associated genetic variation) and its linked gene. Bottom: changes in accessibility and expression following experimental TF knock-out. (B) Schematic of the experimental setup for pooled TF knock-out with multimodal single-cell readouts. Days spanned since thawing of hematopoietic stem and progenitor cells (HSPCs) are noted at the bottom. Between days 0 and 4, cells underwent *in vitro* multilineage differentiation, after which cells underwent *in vitro* erythroid differentiation. (C) Top left: UMAP reduction using RNA measurements collected from all single cells in the experiment. Top right: UMAP reduction using chromatin accessibility measurements collected from all single cells in the experiment. Bottom: UMAP reduction using a weighted-nearest neighbor graph (56) to integrate RNA and chromatin accessibility peak information from the same single cells. Cell types were annotated using a human bone marrow dataset (22) (Materials and Methods). “DC” stands for “Dendritic Cells”, “EoBasoMast Precursor” stands for “Eosinophil-Easophil-Mastocyte Precursor”, “MEP” stands for “Megakaryocytic-Erythroid Progenitors”, “Meg” stands for “Megakaryocytes”, “BFU-E” stands for “Burst-Forming Unit - Erythroid”, and “CFU-E” stands for “Colony-Forming Unit - Erythroid”. (D) Left: normalized Tn5 insertion frequency in BCL11A perturbed cells (perturbation score > 2, Materials and Methods) and control AAVS1 cells. Chromatin accessibility peaks are denoted below and were called using all cells in the experiment. Right: *HBG2* gene expression levels in BCL11A perturbed cells and AAVS1 control cells. *** represents an adjusted p-value < 0.001 using a Wilcoxon Rank Sum test. (E) Left: normalized Tn5 insertion frequency in GATA1 perturbed cells (perturbation score > 2, Materials and Methods) and control AAVS1 cells. Chromatin accessibility peaks are denoted below and were called using all cells in the experiment. Right: *GATA2* gene expression levels in GATA1 perturbed cells and AAVS1 control cells. *** represents an adjusted p-value < 0.001 using a Wilcoxon Rank Sum test.

Perturb-multiome applies a library of CRISPR-based perturbations to a cell population and recovers three distinct readouts from each single cell: (1) the identity of the genetic perturbation (sgRNA), (2) chromatin accessibility at *cis*-regulatory elements (scATAC-seq), and (3) gene expression (scRNA-seq) (fig. 1, A and B).

To apply Perturb-multiome in primary cells, we designed the method to overcome challenges in gene-editing efficiency and to effectively identify a specific perturbation. To achieve high editing efficiency with minimal toxicity, we delivered pooled sgRNAs via an optimized CROP-seq vector and electroporated recombinant Cas9 ribonucleoprotein (6, 18) (fig. 1B, fig. S1 A to C, Materials and Methods). To optimally identify perturbations, we developed a biotin-based enrichment strategy (fig. S11, Materials and Methods, Supplementary Text). Briefly, we achieved high specificity by amplifying CROP-seq transcripts containing the identity of the perturbation in each single cell using biotinylated primers, which were then pulled down with streptavidin-coated magnetic beads and further amplified for sequencing.

We applied Perturb-multiome to primary human HSPCs, which can be differentiated to faithfully recapitulate hematopoietic differentiation *in vitro* (6, 19, 20). We perturbed 19 TFs, ranging from well-known master hematopoietic regulators to others with suggested roles in hematopoiesis (fig. S3A) (21). For each TF, we delivered three different sgRNAs targeting distinct positions in the coding sequences, along with 6 controls (3 non-targeting sgRNAs and 3 sgRNAs targeting the “safe harbor locus” AAVS1 in the genome to account for potential effects induced in cells by double-stranded DNA breaks and repair) (table S1 and Materials and Methods). We profiled cells across four time points. With modest sequencing depth, we detected (i) sgRNA identity in 91.1% of cells (137,604 cells); (ii) an average of 27,774 ATAC fragments and 10,803 unique ATAC peaks per cell; and (iii) an average of 15,970 RNA molecules from 4,129 unique genes per cell (fig. S2, A to H). Across the cell population, we detected 230,083 unique ATACs peaks and 33,415 expressed transcripts.

We focused on the process of red blood cell production, or erythropoiesis, because nearly half of blood cell trait-associated variants have associations with the erythroid lineage, and genetic variation explains a substantial portion (between 7.19–26.95%) of the heritability of these phenotypes (fig. S8A, Materials and Methods) (1, 2, 13). Following a brief initial stage of multi-lineage differentiation to enable some differentiation to non-erythroid fates, we promoted erythroid differentiation of HSPCs. Cells were assayed at days 7, 9, 11 and 14. We recovered a wide range of cell states, from early common progenitors and non-erythroid lineages (megakaryocyte, monocyte, dendritic cell, and granulocyte precursors) to terminal erythroid precursors that closely mimic cell states present in human bone marrow (22) (fig. 1C, fig. S1, D to J, Materials and Methods).

Perturb-multiome faithfully identified various well-characterized gene regulatory mechanisms in hematopoiesis. For example, cells with perturbations in the BCL11A TF, a repressor of fetal hemoglobin and a regulator of the fetal-to-adult hemoglobin switch (23, 24), displayed both increased chromatin accessibility at the fetal hemoglobin promoters and increased gene expression at the fetal hemoglobin genes (*HBG1* and *2*), compared to controls (fig. 1D). Similarly, cells with perturbations in the GATA1 TF showed significantly higher promoter accessibility and gene expression at *GATA2* (fig. 1E), consistent with the negative feedback loop between GATA1 and GATA2 (25, 26). These results illustrate the ability of Perturb-multiome to obtain fine-grained insights into physiologically relevant processes directly in primary human cells.

### Assessment of perturbation effects and identification of transcription factor-sensitive accessible chromatin regions and genes.

As a first step in our analysis, we sought to characterize (1) the gene-editing efficiency of each sgRNA and (2) the perturbation level actually achieved in individual cells, which can vary among cells receiving the same sgRNA.

**Figure 2.**
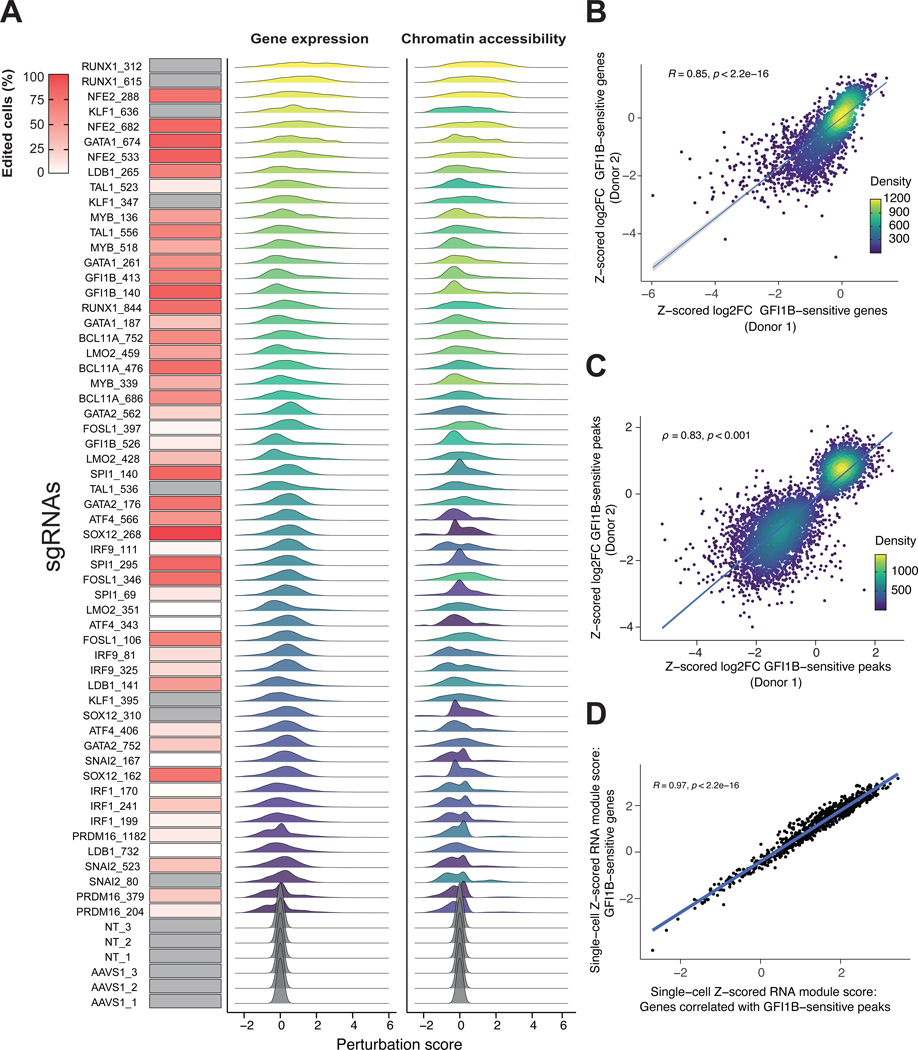
Widespread gene expression and chromatin accessibility changes in response to transcription factor perturbations. (A) Left column:percent of edited cells from pooled single-cell genotyping experiments for each sgRNA (fig. S3, B to D, Materials and Methods). Amplicons with missing data (not covered by single-cell multiplexed PCR) and non-targeting (NT) guides are shown in grey. Middle column: per-guide perturbation score distribution computed on single-cell RNA counts. Right column: per-guide perturbation score distribution computed on single-cell chromatin accessibility peak counts. Each sgRNA is ordered along the color gradient based on the median perturbation score across all cells with that guide, for the RNA or chromatin accessibility modalities. (B) Scatter plot of the Z-scored log_2_FC for RNA gene expression levels (shown are statistically significant genes after multiple hypothesis testing correction) between cells with one of the GFI1B-targeting sgRNAs and AAVS1-targeting control sgRNAs from two independent pooled TF Perturb-multiome screens in two different HSPC donors. The Spearman correlation coefficient ρ is shown, as well as a regression line with confidence intervals. (C) Scatter plot of the Z-scored log_2_FC for ATAC peak counts (shown are statistically significant genes after multiple hypothesis testing correction) between cells with one of the GFI1B-targeting sgRNAs and AAVS1-targeting control sgRNAs for two independent pooled TF Perturb-multiome screens in two different HSPC donors. The Spearman correlation coefficient ρ is shown, as well as a regression line with confidence intervals. (D) Scatter plot of the single-cell Z-scored RNA module score computed using GFI1B-sensitive genes (defined as in panel B) and the single-cell Z-score RNA module score computed using genes correlated with GFI1B-sensitive ACRs (defined as in panel C, Materials and Methods), for cells with a Z-scored perturbation score greater than 1. The Spearman correlation coefficient ρ is shown, as well as a regression line with confidence intervals.

To characterize gene editing efficiency, we used pooled single-cell genotyping (fig. S3B, Materials and Methods) (6). We relied on genotyping rather than the mRNA level of the targeted gene, because some CRISPR-Cas9 edits that disrupt protein-coding regions do not alter mRNA levels. We analyzed 17,665 single cells in droplets, performing 55 multiplexed PCRs in each cell to estimate editing efficiency. These PCRs corresponded to the human genomic sites targeted by sgRNAs, as well as 10 regions within the integrated lentiviral genome to identify the sgRNA in the cell (fig. 2A, left, fig. S3C and D).

Given that a simple binary classification of cells either being perturbed or not has limitations during dynamic processes like differentiation, we defined “perturbation scores” for both the ATAC and RNA data by comparing the chromatin accessibility and gene expression profiles of the cell to those from nearest-neighbor control cells, which represent a similar differentiation state (fig. 2A, middle and right, fig. S3F, Materials and Methods) (27). The perturbation scores were well correlated with sgRNA editing efficiency (fig. S3E). The exceptions included two TFs (GATA2 and SPI1) for which we achieved strong knock-down (confirmed by protein levels), but saw low perturbation scores in erythroid lineages; these TFs have their strongest effects in non-erythroid lineages (fig. S3G) (28, 29). The accessible chromatin regions (ACRs) identified in Perturb-multiome were similar to the ACRs characterized in *ex vivo* bone marrow hematopoietic cells (30), suggesting that our findings are representative of gene regulation in human hematopoietic cells (fig. S3H and I, Materials and Methods).

We then defined TF-sensitive ACRs and TF-sensitive genes by using a linear model that incorporated the perturbation level achieved in each single cell (Materials and Methods). We found that roughly 21.8% of the ACRs (50,114/230,083) (table S2) and 26.0% of expressed genes (8,694/33,415) (table S3) were affected by at least one TF perturbation (fig. S5, A and B). Calibration analyses are included in the Supplementary Text. TF-sensitive and non-TF-sensitive ACRs were similarly distributed across genomic features and the genome (fig. S4B, fig. S8B). Of these TF-sensitive results, 32% of ACRs and 22% of genes responded to only a single TF perturbation (fig. S4, C, D and F). To assess data robustness, we examined an independent biological replicate (using HSPCs from a different donor and sgRNAs targeting different locations in the coding sequences of each TF, for a subset of 8 TFs) and found the results were highly correlated, both qualitatively and quantitatively, with average correlation of >0.9 for TFs targeted in both replicates (fig. 2, B and C, fig. S4A). GATA1-sensitive genes and ACRs were in agreement with independent GATA1 loss-of-function datasets (31, 32) (fig. S7, C and D). In summary, a continuous perturbation score was used to identify TF-sensitive genes and ACRs in the heterogeneous cell populations we studied (Supplementary Text).

### Properties of transcription factor-sensitive ACRs and genes.

**Figure 3.**
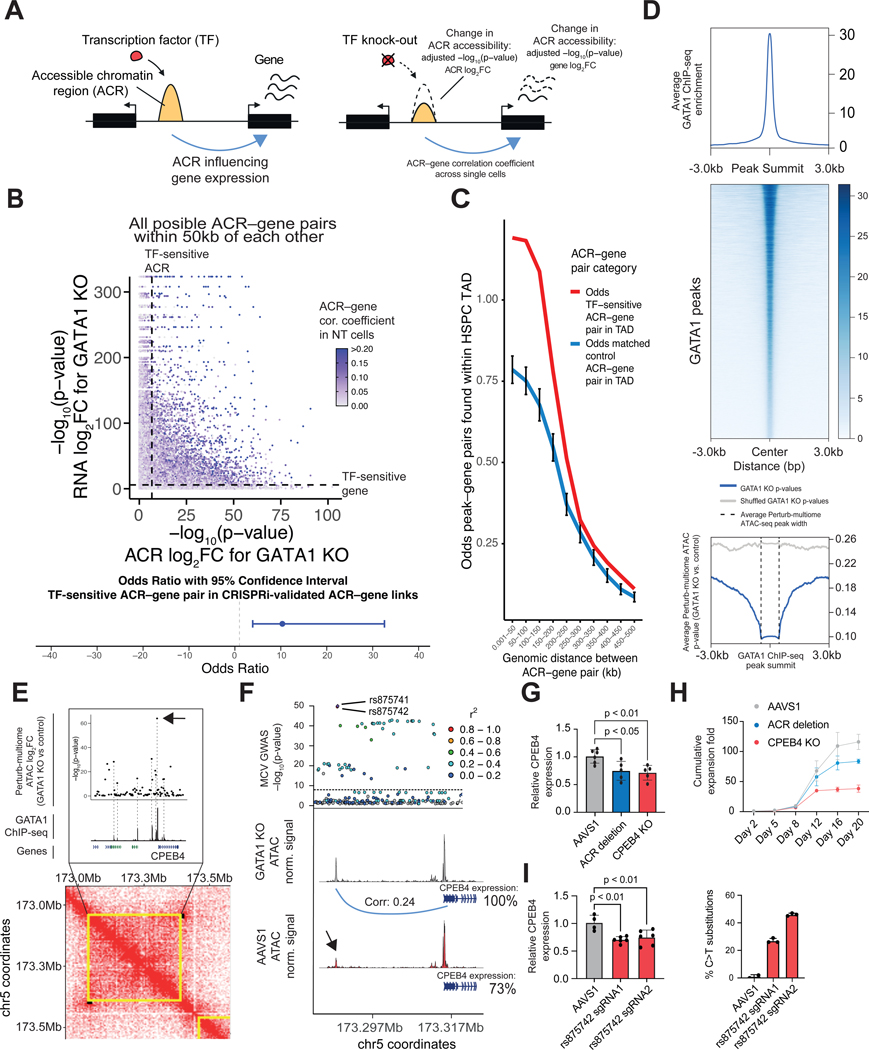
Genome-wide characterization of TF-sensitive elements. (A) Simplified representation of the metrics analyzed in this figure for a basic TF-*cis*-regulatory-element-target gene regulatory network. (B) Top, scatter plots of the ACR and gene –log_10_(*P* value) for GATA1-targeting sgRNAs, respectively, for all possible ACR–gene pairs within 50kb of each other. Dots are colored by the ACR–gene correlation coefficient computed in cells with control NT sgRNAs (Materials and Methods). The vertical and horizontal dashed lines represent the p-value cutoffs for TF-sensitivity of ACRs and genes, respectively. Bottom, odds ratio with 95% confidence interval of a TF-sensitive ACR–gene pair to be found in CRISPRi-validated ACR–gene links (Materials and Methods). (C) Odds of ACR-gene pairs being found within HSPC topologically-associated domains (TADs) (34), as a function of genomic distance and TF-sensitivity status. The odds for TF-sensitive ACR-gene pairs are shown in red. The odds for randomly sampled expression and accessibility matched control ACR-gene pairs (each non-TF-sensitive, each selected among a distribution of non-sensitive elements with similar expression/accessibility levels to their TF-sensitive counterparts) are shown in blue. Each genomic distance bin has the same number of pairs as the TF-sensitive pair, and sampling was repeated 100 times to build a distribution for each genomic bin (34) (Materials and Methods). The bars represent one standard deviation. (D) Top: profile plot of independent GATA1 ChIP-seq enrichment surrounding called ChIP-seq peaks from day 12 erythroid progenitors (36). Middle: heatmap plot for GATA1 ChIP-seq reads surrounding GATA1 peaks. Bottom: profile plot of Perturb-multiome *P* values for the log_2_FC of accessibility for ACRs in cells with GATA1-targeting sgRNAs compared to control centered on GATA1 ChIP-seq peaks as defined in the top panel. (E) Analysis of Perturb-multiome differential accessibility results within an HSPC TAD in hg19 coordinates. Shown are the Perturb-multiome -log_10_(*P* values) for the log_2_FC of accessibility for ACRs in cells with GATA1-targeting sgRNAs compared to control, GATA1 ChIP-seq signal (36), gene annotations highlighting the GATA1-sensitive *CPEB4* gene, and chromosome loops from Hi-C data (34). TADs are shown in yellow and loop anchors in black (Materials and Methods). The TF-sensitive ACR with the most significant change compared to control, discussed in Figure 3F, is highlighted with an arrow. (F) Top: locus zoom plot in hg19 coordinates showcasing -log_10_(*P* values) for variants associated with mean corpuscular volume (MCV) (2). The color scale represents the squared correlation (r^2^) between rs875741 and other neighboring alleles. Middle and bottom: normalized ATAC signal in cells with AAVS1-targeting and GATA1-targeting cells, respectively. The TF-sensitive ACR with the most significant change compared to control is highlighted with an arrow. The Spearman’s rank correlation coefficient (ρ) between this ACR and *CPEB4* is shown, as well as the mean *CPEB4* expression relative to control. (G) Bar plots of the relative *CPEB4* expression for HSPCs edited with a control dual cut in the AAVS1 locus, a deletion of the putative *CPEB4* enhancer on chromosome 5, or a *CPEB4* knock-out, and subsequently differentiated into the erythroid lineage. Data for day 14 of erythroid differentiation, for two distinct HSPC donors. Deletion efficiencies for this experiment are shown in fig. S7G. (H) Cumulative expansion fold for HSPCs edited with a control dual cut in the AAVS1 locus, a deletion of the putative *CPEB4* enhancer on chromosome 5, or a *CPEB4* knock-out, and subsequently differentiated into the erythroid lineage. (I) Bar plots of the relative *CPEB4* expression for HSPCs edited with the TadCBE cytosine base editor precomplexed with AAVS1-targeting control sgRNAs or two different sgRNAs targeting rs875742. Data for day 14 of erythroid differentiation, for two distinct HSPC donors. Right, gene editing efficiencies for one of the donors shown in the left panel.

Having used Perturb-multiome to separately analyze changes in chromatin accessibility and gene expression, we next examined the correlation between these features in single cells carrying a given sgRNA to define ACR-gene linkages (fig. 3A, Materials and Methods). If an ACR regulates a gene and TF perturbation modulates the ACR’s accessibility, the regulated gene will change its expression. To assess ACR-gene linkages, for each cell, we defined two scores: (i) the average expression of the TF-sensitive genes for a given TF and (ii) the average expression of the genes correlated with TF-sensitive ACRs. We found strong concordance between these scores (fig. 2D, fig. S5C and D, Materials and Methods).

First, we examined the effect of TF perturbation on ACR–gene pairs lying within 50 kb of one another. For pairs in which the ACR and the gene were both TF-sensitive (hereafter TF-sensitive ACR–gene pairs), the correlation (at the single-cell level) was higher; this result was seen at all 6 erythroid TFs for which there was a substantial number of such pairs (fig. S6, A and B,fig. S7, A and B). For example, pairs in which the ACR and gene were both GATA1-sensitive had higher correlations across single cells (fig. 3B, top). We found TF-sensitive pairs were 10.34 times more likely among pairs in which the gene responded to enhancer perturbation (by CRISPRi) than among pairs where the gene did not show a response (OR 10.34, p-value < 0.001, Fisher’s exact test) (fig. 3B, bottom, Materials and Methods) (33). This suggests the possibility of using ACR-gene pair TF-sensitivity in correlated pairs to reconstruct gene regulatory networks on a genome-wide scale.

Second, we considered ACR-gene pairs within 2 Mb that showed coordinated changes and examined their three-dimensional proximity, based on Hi-C data from human HSPCs (Materials and Methods) (34). We found that significant TF-sensitive ACR–gene pairs had higher odds of being found within topologically-associated domains (TADs) in HSPCs (OR 1.53, p < 0.001) (fig. 3C).

Third, we observed that TF-sensitive genes exhibited lower RNA polymerase II pausing compared with non-TF-sensitive genes with similar expression levels (fig. S4E, Materials and Methods) (35).

Fourth, we examined whether TF-sensitive ACRs occurred near binding sites for the perturbed TF. We analyzed published ChIP-seq datasets for two of our TFs, GATA1 and NFE2, in primary human proerythroblasts (Materials and Methods) (36). We found that TF-sensitive ACRs in the regions surrounding GATA1 and NFE2 ChIP-seq peaks showed stronger significance levels (that is, higher –log_10_(p-values)) (fig. 3D, fig. S7, E and F).

We then focused on one of the ACR-gene pairs with the highest TF-sensitivity upon GATA1 perturbation. This pair, located at 5q35.2, involves the *CPEB4* gene and an ACR 25 kb upstream of the gene’s TSS and lies within a TAD in HSPCs. *Cpeb4* plays a role in mouse erythropoiesis, but has not been studied in humans (37). The ACR has two variants in strong linkage disequilibrium with each other that are associated with multiple red blood cell traits (1, 2). We found that the most significant TF-sensitive ACRs within the TAD were strongly correlated withGATA1 ChIP-seq peaks (fig. 3E) (2). Besides having high gene-ACR correlation, independently, *CPEB4* and the ACR each also had the most significant changes in response to GATA1 perturbation within the TAD (fig. 3F). These observations suggest that GATA1 binds at the ACR to modulate *CPEB4* expression.

To test this hypothesis, we used CRISPR-Cas9 to delete the ACR in primary cells. The deletion resulted in decreased *CPEB4* expression during erythroid differentiation (fig. 3G, fig. S7G). Furthermore, perturbation of *CPEB4* and, to a lesser extent, deletion of the regulatory element resulted in impaired terminal erythroid differentiation, consistent with the variant associations within the ACR with multiple erythroid traits, and prior studies on the role of *Cpeb4* in mouse erythropoiesis (fig. 3H) (37). We next examined the effect of editing one of the variants with base editor TadCBE (38) (Materials and Methods). This single-nucleotide change reduced the expression of *CPEB4* in primary erythroblasts (fig. 3I). Together, these results illustrate how coupling readouts of TF perturbation, gene expression, and chromatin accessibility in the same single cells can identify TF-sensitive ACR-gene pairs with important roles in human erythropoiesis and can help systematically pinpoint associations between phenotypes and variants discovered by GWAS.

### Transcription factor-sensitive *cis*-regulatory elements are massively enriched in the heritability of blood cell phenotypes

**Figure 4.**
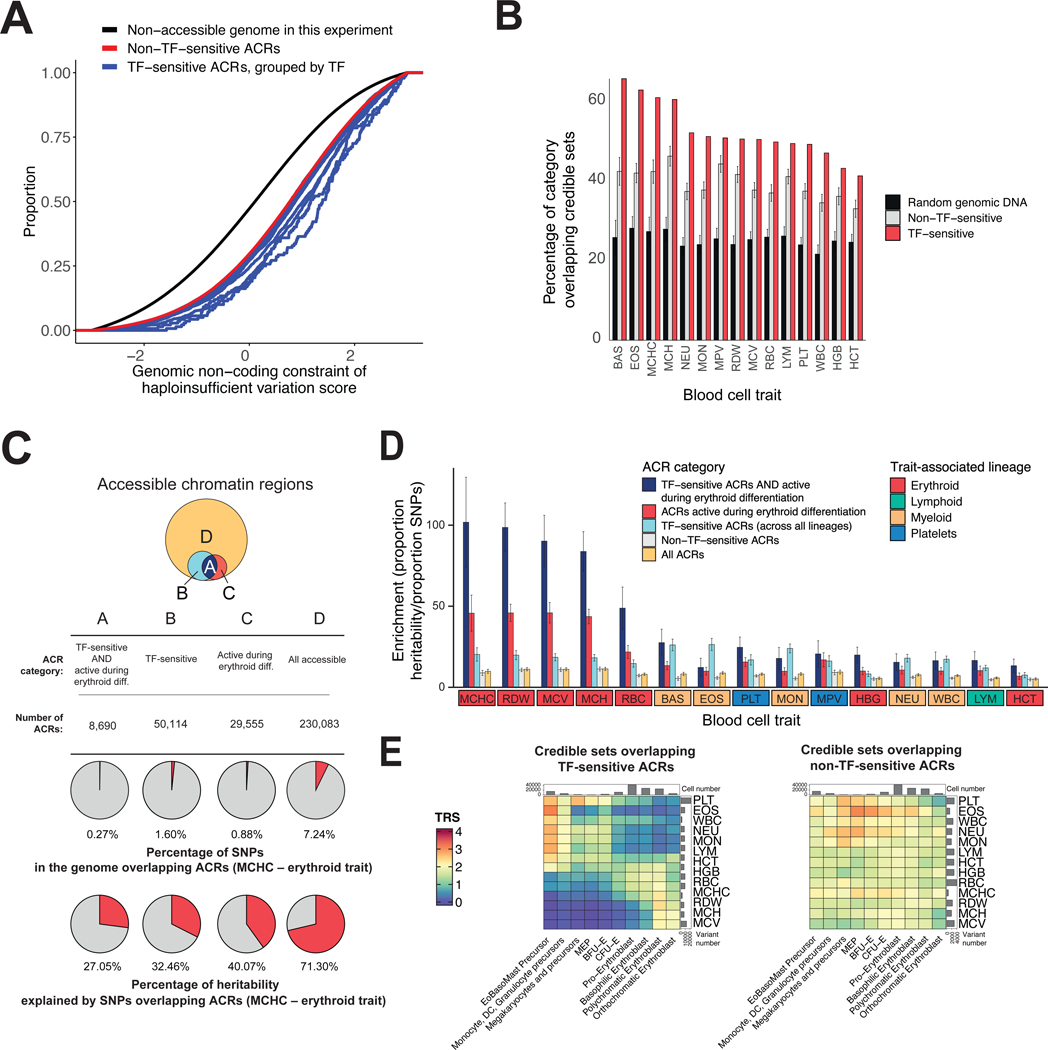
Characterization of genetic variation within TF-sensitive *cis*-regulatory elements. (A) Cumulative distribution of the genomic non-coding constraint of haploinsufficient variation score (39), colored by functional category. Each blue line represents all TF-sensitive ACRs for a given TF. (B) Barplots for the percentage of 95% credible sets overlapping TF-sensitive ACRs, for each blood cell trait. Error bars represent the standard deviation of 100 sampling events of non-TF-sensitive accessible ACRs (“non-TF-sensitive”) or of any genomic region (“random genomic DNA”) (Materials and Methods). (C) Top: area proportional Venn Diagrams representing the number of total ACRs. Middle: percentage of SNPs in the genome that overlap ACRs for mean corpuscular hemoglobin concentration (MCHC). Bottom: percentage of heritability explained by SNPs overlapping ACRs. (D) Barplots of the enrichment (proportion of heritability divided by the proportion of SNPs) for the four functional categories defined in panel C, as well as non-TF-sensitive ACRs. Traits are colored by the respective associated lineage. (E) Heatmaps of the Trait Relevant Score (TRS) (Materials and Methods) for variants belonging to credible sets overlapping TF-sensitive ACRs (left) and not overlapping TF-sensitive ACRs (right). Similar enrichments were observed using only variants from the 95% credible sets directly overlapping with the aforementioned elements (data not shown). Blood cell traits are referred as follows in the figure: BAS = basophil count, EOS = eosinophil count, HCT = hematocrit, HGB = hemoglobin, LYM = lymphocyte count, MCH = mean corpuscular hemoglobin, MCHC = mean corpuscular hemoglobin concentration, MCV = mean corpuscular volume, MON = monocyte count, NEU = neutrophil count, PLT = platelet count, RBC = red blood cell count, RDW = red cell distribution width, WBC = white blood cell counts. BAS, EOS, MON, NEU, WBC are non-erythroid myeloid traits; LYM is a lymphoid trait; PLT is a megakaryocytic trait; HCT, HGB, MCH, MCHC, MCV, RBC, RDW are erythroid traits.

Encouraged by the *CPEB4* example, we sought to examine more broadly how TF-sensitive regulatory regions were related to naturally occurring genetic variation underlying blood-cell phenotypes and diseases. We began by noting that TF-sensitive ACRs are under greater constraint (lower rate of genetic variation) (39) compared to the non-accessible genome (fig. 4A, Materials and Methods). Furthermore, for several TFs, the average constraint for TF-sensitive ACRs was higher than for non-TF-sensitive ACRs. This suggested that the TF-sensitive ACRs may play key, conserved functional roles in hematopoiesis.

We explored whether genetic variants associated with blood-cell phenotypes were enriched in the TF-sensitive ACRs, by examining how credible sets for each trait overlapped with TF-sensitive ACRs, non-TF-sensitive ACRs, and random genomic regions. We found that, averaged across traits, the proportion of regions that contained a variant in a credible set for a trait was much higher for TF-sensitive ACRs (50.59%) than for non-TF-sensitive ACRs (39.25%) and random genomic regions (22%) (fig. 4B). When we examined the results for individual TFs and traits, we found striking differences. The highest proportion was 65.75% for TF-sensitive ACRs containing variants in credible sets for basophil number (fig. 4B, fig. S8F, fig. S9, Materials and Methods).

For each of the blood cell traits, we also examined the proportion of heritability explained by SNPs in various regions, using partitioned linkage disequilibrium score regression (LDSC) (Materials and Methods). We examined four types of ACRs: (i) TF-sensitive ACRs active during erythroid differentiation, (ii) all TF-sensitive ACRs, (iii) all ACRs active during erythroid differentiation, and (iv) all ACRs observed in both erythroid and non-erythroid cells, (Materials and Methods, fig. S8C). While TF-sensitive ACRs active during erythroid differentiation comprise <0.3% of the genome, heritability in these regions showed ~100-fold enrichment for several traits — that is, 22.25% of the heritability for erythroid traits lies in this small proportion of the genome (fig. 4, C and D). In contrast, the ACRs active during erythroid differentiation and all TF-sensitive ACRs showed lower enrichment (up to ~46-fold and ~26-fold enrichment for certain traits, respectively) (fig. 4, C and D). We observed similar trends for posterior probability distributions of fine-mapped variants (fig. S10). The set of all ACRs comprises much more of the genome (7.24%) and explains more of the heritability (~79%), but the enrichment is much lower (~11-fold) (fig. 4, C and D). Notably, the TF-sensitive ACRs active during erythroid differentiation were also significantly enriched in TF binding motifs compared to all ACRs active during erythroid differentiation (fig. S8, D and E). Furthermore, we observed that credible sets of GWAS variants that lie within the TF-sensitive regions showed clear patterns of cell-type specific enrichments for their respective traits, unlike other variants (fig. 4E, Materials and Methods). These results suggest a critical role for TF-sensitive ACRs and the associated variants lying within them in explaining the heritability of blood cell phenotypes.

Collectively, we find that ACRs that change in response to TF perturbation are disproportionately enriched for phenotype-relevant genetic variation. More broadly, if this is true for other complex diseases and phenotypes, it suggests that systematic mapping of TF-mediated gene regulatory networks could accelerate variant-to-function mapping.

## Discussion

Understanding the molecular and functional consequences of non-coding variation is essential to dissect the mechanisms underlying complex diseases and phenotypes. These insights have proven key for the development of new diagnostic and therapeutic interventions, as recently achieved in hemoglobin disorders by emerging gene therapies (11, 20, 36, 37, 39). Scalable approaches to functionalize regulatory elements and link them to large sets of candidate phenotype-associated genetic variation are critical for making progress towards these goals, especially in human tissues with complex mixtures of cells.

Analysis of genome-wide TF-sensitive regions after perturbation of 19 TFs identified a tiny subset of the genome (<0.3%) highly enriched in heritability for blood cell phenotypes, relative to hematopoietic- or erythroid-specific accessible chromatin. Notably, these regions overlapped an average of 51% of credible sets for blood cell traits (ranging 41.31–65.75%), and had up to a 100-fold heritability enrichment for certain cell-traits. In the future, expanding perturbations to more TFs will likely uncover additional enrichments for genetic variation.

Several of the TFs studied are master regulators of hematopoietic differentiation and their perturbation results in developmental blocks (40–43). We nonetheless obtained fine-grained observations of the effects of TFs at each stage by using analytical approaches that weigh comparisons by the perturbation scores of each single cell with respect to nearest-neighbor control cells, thereby leveraging the asynchronous nature of hematopoietic differentiation and overcoming the variable efficiencies and effects of perturbations.

The TF perturbation approach is modular and compatible with alternative strategies to modulate gene expression (44, 45). Endogenous tagging of TFs with degrons or related approaches could explore the immediate consequences of TF degradation (46–49). However, this strategy is not currently applicable to large numbers of regulators and in HSPCs isolated from human donors (50). While we focused on single perturbations per cell, future efforts using multiplexed perturbations could be used to discern the combinatorial logic of TF regulatory control (51, 52). While this work was under review, additional studies reported distinct, but related approaches to perform pooled perturbations with multiomic readouts in cell lines (53, 54), illuminating the broad potential for these types of approaches and the possibility to expand them with additional genome perturbation tools.

We focused on the study of TF-sensitive regions and likely causal variants, which allowed us to study a larger fraction of phenotype-relevant variation beyond those that directly disrupt TF-binding motifs (13). These regions will be excellent candidates for saturation mutagenesis to identify functional base pairs and pinpoint causal variants. In addition, TF-sensitive networks are likely to harbor additional genes and associated regulatory elements with still-to-be characterized critical hematopoietic functions.

We envision that the identification of TF-sensitive gene regulatory networks through perturbation approaches will help in prioritizing and understanding the mechanisms underlying a wide range of human diseases and phenotypes across many tissues and organs.

## Supplementary Material

Supplementary Material

Table S1

Table S3

Table S2

Table S4

Table S5

Table S6

## Data Availability

Raw and processed data have been deposited at GEO (accession numbers GSE274110 and GSE274113) and the corresponding code is available in our GitHub repository (https://github.com/sankaranlab/perturb_multiome) and Zenodo (55).
