# Supplementary material for: Transcription factor networks disproportionately enrich for heritability of blood cell phenotypes
Source: Science. Author manuscript; Available in PMC 2025 Jun 16. (PMC12168499; doi:10.1126/science.ads7951)
Supplement: Supplementary Material [file NIHMS2076308-supplement-Supplementary_Material.docx]

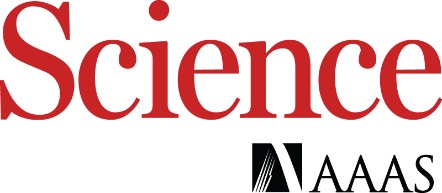


Supplementary Materials for

**Transcription factor networks disproportionately enrich for heritability of blood cell phenotypes**

Jorge D. Martin-Rufino, Alexis Caulier, Seayoung Lee, Nicole Castano, Emily King, Samantha Joubran, Marcus Jones, Seth R. Goldman, Uma P. Arora, Lara Wahlster, Eric S. Lander, Vijay G. Sankaran

Corresponding authors: sankaran@broadinstitute; acaulier@broadinstitute.org

**The PDF file includes:**

Materials and Methods

Supplementary Text

Figs. S1 to S14

References (*56 - 69*)

**Other Supplementary Materials for this manuscript include the following:**

Tables S1 to S6

Materials and Methods

**Lentiviral vector**

We employed a modified CROP-seq vector for efficient single-cell perturbational screens in hematopoiesis that we previously described (*6*). The vector has been deposited in Addgene (<https://www.addgene.org/203321/>).

**Lentiviral production**

Lentiviral particles were produced as previously described (*6*). Briefly, Human Embryonic Kidney-293T cell line (ATCC, CRL-3216), was cultured in DMEM Life Technologies, 1965-118) supplemented with 10% FBS (BioTechne, S11550) and 1% penicillin-streptomycin (Life Technologies, 15140-122). When they reached ~70% confluency, cells were co-transfected with the pooled CRISPR library along with VSV-G and pDelta 8.9 packaging vectors overnight, using FuGene lipofection (Promega, E2691). Media was changed the following morning to DMEM supplemented with 20% FBS, and viral supernatant was first collected 24 hours later and kept at 4ºC. Media was subsequently replaced and harvested again 48 hours later. After cold centrifugation at 1,500 rpm and filtration through 0.45µm PVDF membrane (Millipore, SCHVU01RE) to remove cell debris, supernatants were pooled and ultracentrifuged at 24,000 rpm, 4ºC for 1:30h (Beckman Coulter SW32Ti). The pellet was ultimately resuspended with StemSpanTM SFEM-II media (Stemcell Technologies, #09655) and stored at -80ºC until further usage.

**Primary cells**

CD34+ primary human hematopoietic frozen cell samples from XY healthy donors were purchased from Fred Hutchinson Cancer Center in 5 million vials and processed as described below.

**Erythroid culture**

5 million CD34^+^ cells were thawed in 1X PBS (Gibco, 10010-023) supplemented with 1% FBS, centrifuged at room temperature, 350xg for 5min, and resuspended in 10mL of hematopoietic stem cell (HSC) expansion media consisting of StemSpanTM SFEM-II media (Stemcell technologies, #09655) supplemented with 1% L-Glutamine (Thermo Fisher Scientific, 25-030-081), 1% penicillin/streptomycin (Life Technologies, 15140-122), 50ng/mL human TPO (PeproTech, 300-18) and 100x CC100 cytokines (StemCell Technologies, 02690). Erythroid differentiation was then induced in 3 steps as previously described (*6*). On day 3, cells were washed in 50mL 1X PBS and resuspended in phase I erythroid media at a density of 10^5^ cells/mL. Phase I media is IMDM based (Life Technologies, 12440-061) and supplemented with 3% AB human serum (Atlanta Biologicals, S40110), 2% AB human plasma (SeraCare, 1810-0001), 1% penicillin/streptomycin (Life Technologies, 15140-122), 10µg/mL recombinant insulin (Lilly, NDC 0002-8215-01), and 3 IU/mL heparin (Hospira, NDC 00409-2720-01), 200µg/mL human holo-transferrin (Sigma-Aldrich, T0665), 1ng/mL recombinant human IL-3 (Peprotech, 200-03), 10ng/mL human recombinant Stem Cell Factor (SCF, Peprotech, 300-07), 3IU/mL recombinant human erythropoietin (EPO, epoetin alpha, Amgen, NDC 55513-267-10). Cells were spinfected with the CRISPR library on day 2 overnight, then washed and resuspended in the same phase I media. Cells were nucleofected with Cas9 on day 4 and resuspended in phase I media until day 7. On day 7, recombinant human IL-3 was withdrawn from the media and cells were cultured until day 11 in phase II media. On day 11, holo-transferrin concentration was increased to 1mg/mL and both IL-3 and SCF were removed from the media, and cells were cultured in phase III media until the end of the culture. Media was freshly renewed every 2-3 days, and cells were thoroughly washed in 1X PBS (Gibco, 10010-023) to remove the cytokines at each step of the culture.

**Lentiviral transduction**

On day 2, CD34+ cells were infected at a density of 10^6^ cells per mL in HSC expansion media supplemented with 8µg/mL polybrene (Sigma Aldrich, TR-1003-G). Freshly thawed lentiviral particles were added at a MOI of 0.3-0.5, and cells were spinfected at 2,000 rpm for 1:30h at 37ºC. Cells were kept overnight at 37ºC. The next morning, infected cells were washed in 50 times their volume of 1X PBS and resuspended in Phase-I erythroid media.

**Cas9 electroporation**

Cells were nucleofected on day 4, 2 days after spinfection, after verifying the expression of VEX reporter by flow cytometry. To nucleofect 15 million cells, 10µl of 62µM Alt-R S.p. HiFi Cas9 Nuclease V3 (IDT, 1081061) was diluted in 21µl of 1X PBS and then combined with a non-targeting sgRNA in a swap strategy that has proven to increase the editing efficiency in HSCs for cutting Cas9 (*18*), for 20min at room temperature. In the meantime, cells were washed twice with 1X PBS to remove RNAses from the media, then resuspended in 190µl P3 solution with supplement (Lonza, VPA-1003) and 10µl of 100µM Alt-R Electroporation enhancer (IDT, 1075916). Cell suspension was then dispensed in two 100µl electroporation cuvettes and electroporated in a 4D-Nucleofector X Unit (Lonza) using the DZ-100 program. Immediately after, 500µl of phase-I erythroid media was added to each cuvette and incubated at 37ºC for 5min. Then, cells were resuspended in phase-I media at a density of ~500,000 cells per mL.

**FACS enrichment prior to single-cell genomics**

To enrich cells expressing a single gRNA at each time point (days 7, 9, 11 and 14), we sorted cells for expression of VEX (Violet light excited GFP reporter present in the lentiviral vector, **fig. S1C**). 10mL of cell suspension was washed in 40mL of 1X PBS (x300g, 5min, room temperature) and the pellet was resuspended in 2mL 1X PBS + 0.04%BSA. 6-8x10^5^ cells were sorted in a 5mL low binding tube (Eppendorf, 0030122356) pre-coated with 1mL of 1X PBS + 0.04%BSA (Sigma-Aldrich, A9418), using a Sony MA900 sorter. Sorted cells were then pelleted at 300g for 5min, at 4ºC, resuspended in 250µl of 1X PBS + 0.04%BSA and placed on ice.

**Multiparametric flow-cytometry (FCM) of cell differentiation**

At each time point, 10^5^ unsorted cells were washed in 1mL of 1X PBS and resuspended in 100µl of 1X PBS + 0.04%BSA. 5x10^4^ cells were used for unstained and each single cell color control. Cells were stained with a cocktail of antibodies targeting CD123, CD36, CD34, CD71 and CD325a, and 7-AAD or with one of those as a single-color control (**table S4**). On day 14, only antibodies targeting CD71, CD235a and 7-AAD staining were used. After 30min of incubation at room temperature in the dark, cells were washed twice with 1mL 1X PBS + 0.04% BSA, and resuspended in 100µl of the same solution. Acquisition was performed on the same Sony MA900 as sorted cells. For intracellular staining, 10^5^ cells were stained with anti-human CD71 and CD235a antibodies, and fixed in 1X PBS + 4% paraformaldehyde (PFA) for 15min at room temperature. After 2 washes in 1X PBS, cells were resuspended in 1X PBS + 2% FBS. To permeabilize, 100% Methanol was added to a final concentration of 90% while gently vortexing and incubated on ice for 10min. After 2 washes, cells were incubated for 30min in the dark at room temperature in 1X PBS + 2% FBS with intracellular antibodies targeting PU.1, GATA-1, or GATA-2 at manufacturer’s recommended dilution. Cells were washed twice and fluorescence was read on a CytoFLEX S cytometer (BD).

**Morphological analysis of erythroid differentiation**

At each time point (days 7, 9, 11 and 14), ~10^5^ sorted cells were washed and resuspended in 150µl of 1X PBS + 50% FBS and centrifuged in a Cytospin 4 centrifuge (Thermo Fisher Scientific) at 500rpm for 5min, with low acceleration. Slides were air dried and first stained with May-Grünwald solution (Sigma Aldrich, MG1L) for 5 min, rinsed 4 times in MilliQ water for 30s per wash, then stained with 1:20 dilution of Giemsa solution (Sigma Aldrich, 32884) for 15min and rinsed 6 times in MilliQ water for 30sec per wash. Slides were air dried overnight and imaged on a Mica instrument (Leica microsystems).

**Precision nuclear run-on sequencing (PRO-seq)**

CD34+ cells from 2 different XY donors were cultured in erythroid phase I media for 8 days, and electroporated with 1nmol GATA1 or AAVS1 control sgRNAs pre-complexed with 1nmol Cas9 15min at RT. 5-10 million cells were electroporated in P3 solution with P3 Primary Cell 4D-Nucleofector™ LV Kit L, program DZ-100 (V4LP-3002), and resuspended in phase II erythroid media. Editing efficiency was 80% as assessed by Sanger sequencing, and flow cytometry showed a 3-fold decrease in intracellular GATA1-PE mean fluorescence intensity in the CD71^+^/CD235a^-^ progenitor population. Cells were then harvested 24h post-electroporation and processed following the Harvard Nascent Transcriptomics Core cell permeabilization protocol for PRO-seq (*58*). Briefly, cells were harvested on ice in 5mL low binding tubes (Eppendorf, 0030108310), and washed twice in cold PBS at 400g, 4ºC, for 5min. After removing cold PBS, cells were mixed in 200µl of Wash Buffer (W) to resuspend a single cell suspension. Cells were permeabilized in 4mL of Permeabilization Buffer (P) for 5min on ice. Cells were then centrifuged at 400g, 4ºC, for 5min, to remove the supernatant and immediately resuspended in 1mL of W-buffer to dilute any remaining P-buffer. After gently mixing, 4mL of W-buffer was added and cells were washed at 400g, 4ºC, for 8min. The supernatant was removed and cells were resuspended in 2x100µl F-buffer in a 1.5mL low binding tube (Eppendorf, 022431021). Cells were counted and Freezing Buffer (F) was adjusted to a cell concentration of 5-10x10^6^/mL, flash frozen on dry ice. PRO-seq was then performed as previously published (*59*).

**Assessment of enhancer cutting efficiency**

Genomic DNA was extracted from ~100,000 cells using QuickExtract™ DNA Extraction Solution (Biosearch Technologies, QE0905T). The chromosome 5 enhancer locus was PCR amplified using Platinum™ II Hot-Start PCR Master Mix (2X) (ThermoFisher scientific, 14000013) for 30 cycles, and its deletion was verified on E-Gel™ 2% Agarose gel with SYBR™ Safe DNA stain (ThermoFisher scientific, A42135). Relative quantification of fragments was assessed with the Agilent High Sensitivity DNA Kit ([5067-4626](https://www.agilent.com/store/productDetail.jsp?catalogId=5067-4626&catId=SubCat2ECS_227709)) on a Agilent 2100 Bioanalyzer instrument. The 900bp/1,650bp molarity ratio was estimated using the bioanalyzeR R package. Deletion efficiency was confirmed with Sanger sequencing on the column-purified PCR fragments (QIAquick PCR Purification Kit, Qiagen, 28104), using the forward PCR primer for sequencing. Primers are referenced in **table S5**.

**Real-Time quantitative Polymerase Chain Reaction (RT-qPCR)**

Total RNA was extracted from around 100,000 cells using column purification with removal of genomic DNA using DNAse I (RNEasy Micro Kit, Qiagen, 74004). After Reverse Transcription (iScrpit^TM^, BioRad, 1708841), 10ng of complementary DNA (cDNA) was used for SYBR green quantitative PCR (LightCycler® 480 SYBR Green I Master, 04887352001) and detected on CFX384 Touch Real-Time PCR Detection System, BioRad). Primers are listed in **table S5**.

**Cytosine base editing**

The TadCBE cytosine base editor was purified using the procedure we previously reported (*6*). We designed a codon optimized version of TadCBE with an N-terminal His tag (*38*). The plasmid for purification has been deposited in Addgene (https://www.addgene.org/225093/).

We performed two sequential electroporations, on day 3 and day 4 after thawing, using the DZ-100 electroporation program in a 4D-Nucleofector X Unit (Lonza), as previously described for HSPC electroporation with base editors (*6*). Immediately after, 100µl of phase I erythroid media was added to each cuvette and incubated at 37ºC for 5min. Then, cells were distributed in phase I media at a density of ~500,000 cells per mL. Cuvettes were again washed with 75µl of phase I erythroid media to retrieve any cells from the cuvette.

**Perturb-multiome**

At each time point, 160,000 sorted cells were processed in 4 technical replicates. Nuclei were extracted following 10x Genomics recommendations for nuclei isolation for single cell multiome CG000365 RevB, with the recommended buffers. All centrifugation steps were carried in a swinging bucket centrifuge to reduce cell loss. 40,000 sorted cells were placed in a low binding tube (Eppendorf), volume was adjusted to 50µl with 1X PBS + 0.04% BSA, and cells were centrifuged at 300 rcf for 5min at 4ºC. 45µl of supernatant was removed and replaced with 45µl of pre-chilled Lysis buffer containing 10mM Tris-HCl pH 7.4, 10 mM NaCl, 3 mM MgCl2, 1% BSA, 0.1% Tween 20, 0.1% NP-40, 0.01% digitonin, 1 mM DTT, 1 U/mL Protector RNase inhibitor, and gently mixed 3 times before incubating 3 min on ice. Then, 50µl of chilled wash buffer containing 10 mM Tris-HCl pH 7.4, 10 mM NaCl, 3 mM MgCl2, 1% BSA, 0.1% Tween 20, 1 mM DTT, 1 U/mL Protector RNase inhibitor, was added to each tube without mixing, and tubes were centrifuged at 500 rcf for 5 min at 4ºC. 95µl of supernatant was then removed and replaced with 45µl of pre-chilled diluted nuclei buffer containing 1X final concentration of 20X Nuclei Buffer, 1 mM DTT, 1 U/mL Protector RNase inhibitor. Nuclei were centrifuged at 500 rcf for 5 min at 4ºC, and supernatant was carefully removed in totality. Nuclei pellet was resuspended in 7µl of the same chilled nuclei buffer, and 1µl of the nuclei suspension was stained with 1:10 dilution of 1 mg/mL Propidium Iodide (Life Technology, P3566) and manually counted on a hemocytometer (INCYTO, DHC-N01-2). Nuclei quality was assessed visually on a Nikon Eclipse TS100 microscope after diluting 1µl of the nuclei suspension in 4µl of chilled nuclei buffer and stained with 5µl of 0.4% Trypan blue solution (Invitrogen, T10282). Immediately after counting, 16,100 nuclei per replicate were processed into multiome protocol to target 10,000 nuclei recovery. Transposition, GEM generation and barcoding, and libraries construction were prepared following 10x Genomics protocol for Chromium Next GEM Single Cell Multiome ATAC + Gene Expression CG000338 Rev E. The four replicates were processed in parallel at each time point, in the same Next GEM Chip J (10x Genomics, PN-1000230). The GEM emulsion was then stored at -80ºC for a few days until all time points were harvested, and the 16 samples were then processed together for clean-up and further steps. The validation experiment was processed exactly in the same way, but only 2 replicates per time point were processed in parallel.

**Pooled single-cell genotyping**

To determine the editing efficiency at single cell level, we designed primer pairs to amplify ~300bp genomic regions around the predicted cutting site of each sgRNA, as well as 9 primers targeting regions of the integrated CROP-seq vector **(fig. S3B, table S5)**. All PCR reactions are multiplexed in each individual cell, and we obtained a panel uniformity (defined as percentage of amplicons with >0.2x reads above the mean) of 83.64%. Two days after thawing, 2 million CD34+ XY cells were spinfected with the pooled-CRISPR library at a MOI ~0.3-0.5 following the method described in the corresponding section of the manuscript and incubated overnight at 37ºC. Cells were washed the next morning then resuspended in phase-I of erythroid media. On day 5, 8 million cells were electroporated with Cas9 ribonucleoprotein complexed with a non-targeting sgRNA, following the protocol further detailed. Cells were then resuspended in phase I of erythroid media until day 8. On day 8, 900,000 Vex+ cells were FACS sorted following the protocol detailed in the corresponding section of the manuscript and concentrated to 3,000 cells /µl in 1X PBS + 0.04% BSA and placed on ice. 2 replicates of 100,000 cells were immediately processed using the Tapestri single-cell DNA sequencing V3 (Mission Bio) following manufacturer’s recommendations (User Guide QRC v3_MB05-0010_rev2).

**CROP-seq transcript enrichment**

A step-by-step protocol has been included in **Supplementary Text**, which also includes a graphical summary of the protocol**.** To improve the detection of sgRNAs at single cell level, we amplified CROP-seq transcripts containing the sgRNA information using a 2-steps polymerase chain reaction (PCR), from the cDNA amplified in 10x multiome workflow. First, 10ng of cDNA from each sample was amplified with NEBNext® Q5U® Master Mix (NEB, M0597S), with a biotinylated forward primer (/5Biosg/UAUAGTGACTGGAGTTCAGACGTGTGCTCTTCCGATCTCGATTTCTTGGCTTTATATATCTTGTG) and a modified phosphorothioate reverse primer to protect from nuclease cleavage (CTACACGACGCTCTTCCGAT*C*T), with the following parameters: 98ºC for 30s, then 8 cycles at 98C for 15s for denaturation, 69ºC for 15s for primer annealing, 72ºC for 20s for fragment elongation, and a terminal elongation step at 72ºC for 120s. PCR1 fragments were cleaned up with 1X SPRIselect beads (Beckman Coulter B23318) and eluted in Buffer EB (Qiagen, 19086). Then, biotinylated PCR1 fragments were enriched by Dynabeads™ MyOne™ Streptavidin C1 (Thermo Fisher scientific, 65001) following manufacturer’s recommendations. Briefly, after 4 washes in WB buffer, PCR fragments were incubated with 0.5X pre-washed Dynabeads™ for 15min at room temperature, on a rotating mixer (HulaMixer, Thermo Fisher scientific), washed 3 times in WB buffer, and incubated in a master mix of 15uL Tris-EDTA buffer solution pH 7.4 (Sigma Millipore, 93302) and 0.5uL of Thermolabile USER (Uracil-Specific Excision Reagent) II Enzyme (NEB, M5508S) for 30min at 37ºC on a rotating mixer. PCR1 fragments were then cleant-up with 1X SPRIselect beads and eluted in buffer EB for the second PCR. PCR2 primers included universal Illumina sequencing adaptors P5 and P7 with a different index for each sample, and amplification was carried out with Q5® High-Fidelity DNA polymerase (NEB, M0492L) as following: initial denaturation at 98ºC for 30s, followed by 26-30 cycles (determined by qPCR) at 98ºC for 15s for denaturation, 69ºC for 15s for primer annealing, 72ºC for 20s for elongation, and a terminal elongation step at 72ºC for 120s. PCR2 fragments were finally cleaned-up with 1X SPRIselect beads and resuspended in buffer EB for sequencing. Primer sequences are references in **table S5**.

**Sequencing**

Quantification and quality of all libraries were assessed on a Bioanalyzer 2100 using High-Sensitivity DNA Reagent Kit (Agilent, 5067-4626). Single-cell gene expression and ATAC replicates were separately pooled to 2nM, and final concentration was assessed by qPCR using KAPA-quantification kit (Roche, KK2602). Both libraries were sequenced using a Novaseq 6000 S4 kit (Illumina) in pair-ended mode. The CROP-seq transcript enrichment library was sequenced with a NextSeq 1000/2000 P3 100 cycles (Illumina) in pair-ended mode, with a custom primer for read 2 (CGATTTCTTGGCTTTATATATCTTGTGGAAAGGACGAAACACCG). The single cell genotyping library was sequenced with a NextSeq 1000/2000 P2 300 cycles (Illumina) in pair-ended mode.

**Perturb-multiome data processing**

Custom scripts for the analyses described in the subsequent sections can be found in this paper’s GitHub repository, available at <https://github.com/sankaranlab/perturb_multiome> and Zenodo *(55)*.

Raw bcl files were demultiplexed using bclconvert v4.0.3. Gene expression and ATAC libraries were processed using Cellranger ARC 2.0.2. CROP-seq guide enrichment libraries were processed jointly with gene expression libraries using Cellranger v7.0.1. Matrices from all modalities were merged downstream in R. Using Seurat v4, standard processing of scRNA-seq data was performed with *FindVariableFeatures, ScaleData, RunPCA, FindNeighbors, FindClusters* and *RunUMAP* (*56*)*.* For the ATAC data, standard processing using Signac and *RunTFIDF, FindTopFeatures, RunSVD* and *RunUMAP* was performed (*60*). To create the weighted nearest neighbors graph, *FindMultiModalNeighbors* was used and then UMAP was run on the joint space. Cell types were annotated using human bone marrow data (*22*).

**Pooled single cell data processing**

Sequenced fastq.gz files were demultiplexed using bclconvert v.4.0.3 and processed using the Tapestri Pipeline v3, which comprises adapter trimming, alignment, cell barcode error correction, cell identification among droplets and variant calling, followed by MissionBio’s Genome Editing Solution.

Mutational frequencies in **fig. S3D** are a weighted frequency of the times that a given type of mutation was seen across reads for one cell barcode, which was then averaged across cells.

**Perturbation score calculation**

Additional details regarding the method are provided in the **Supplementary Text**. An initial set of differentially expressed genes or differentially accessible chromatin regions was identified by comparing cells with a given guide to non-targeting cells (following mitigation of shared variation by subtracting the average expression or accessibility profiles of control neighbor cells, in a similar differentiation state, from the profile of cells with a given targeting sgRNA) using Mixscale (*27*). This defines a Z-scored perturbation score for each single-cell. That score was then used as a weight in a linear regression model to predict target gene expression levels, such that cells with higher perturbation scores are given a higher weight. In the end, if a perturbed gene controls a target gene, the term associated with that score in the model will be significantly different from 0. To avoid circularity in this strategy, a leave-one-out approach was used, in which the gene predicted in the regression is taken out from the initial perturbation score computation.

We extended this approach to the ATAC dimension by modeling data as fragment counts, rather than reads (*61*), and using the LSI reduction to calculate perturbation signatures.

TF-sensitive elements were selected for each transcription factor as those whose p-values were significant following Bonferroni correction for multiple hypothesis testing.

**Perturb-multiome ACR comparison with ACRs from *ex vivo* bone marrow hematopoietic dataset**

The *ex vivo* dataset includes pooled single-cell multiome data of bone marrow hematopoietic cells freshly harvested from 4 healthy donors (*30*). Cell types were annotated using the transcriptional profiles with an available high-resolution single cell atlas of human hematopoietic bone marrow cells (*22*). The *ex vivo* dataset was subset to keep cells of similar lineages and differentiation stages to those present in our *in vitro* dataset. 50 ACRs markers per cell state were identified using Seurat’s FindMarkers() on the *ex vivo* dataset. For each set of cell state markers, an ATAC signature score was calculated for individual cells in our *in vitro* dataset, using the AddModuleScore() function from Seurat. The values were then averaged per cell group and presented as a heatmap.

**PRO-seq analysis**

Sequencing reads were trimmed to 41bp, and only those with an average quality >20 were retained. Adapters were removed using cutadapt 1.14 and 3’ bases with low quality using –match-read-wildcards -m 20 -q 10. An initial alignment step of R1 reads was performed to the control Drosophila genome spike-in using Bowtie 1.2.2 (with the parameters -v 2 -p 6 --best --un). Unmapped reads were then aligned to the hg38 genome (-v 2 --best), sorted and converted to bedGraph format.

RNA polymerase pausing analysis was performed after counting read counts surrounding active dominant transcription start sites (TSSs) using proTSS (<https://github.com/NascentTranscriptionCore/proTSScall>) compared to gene bodies. Dominant TSSs and dominant transcription end sites (TESs) were obtained from this 5’ PRO-seq data and our previously published human erythropoiesis RNA-seq dataset (*6*).

**Gene–ACR correlation analysis**

Gene expression and chromatin accessibility correlations were computed across single cells for each sgRNA using *FigR* (*62*)*.* Briefly, *FigR* calculates the gene-ACR Spearman correlation across single cells and obtains significance estimates by permuting the correlation coefficients of a set of GC-matched background ACRs. We computed correlations over 50kb or 2Mb for every gene-ACR pair.

**Analysis of the overlap between TF-sensitive genes and genes correlated with TF-sensitive ACRs**

We calculated the average expression levels of genes of interest, subtracted by the aggregated expression of control genes with similar expression, using the *AddModuleScore* function from Seurat (*56*). We did this for TF-sensitives and for genes correlated with TF-sensitive ACRs. Genes correlated with TF-sensitive ACRs were defined as described in the section “Gene–ACR correlation analysis” above, using gene–ACR pairs with a pvalZ < 0.1 and with a correlation coefficient > 0.03 in NT cells (*62*).

**Analysis of the overlap between TF-sensitive ACR–gene pairs and CRISPRi-validated ACR–gene pairs**

To assess ​​how well ACR–gene pairs reflect experimentally established links (e.g., those from ACR-CRISPRi experiments), we used validated enhancer-gene pairs established in K562 cell lines by CRISPRi. These pairs were used by ENCODE-r2EG as ground truth to train their enhancer-gene model (*33*). This dataset contains 10,411 ACR-gene pairs, of which 472 were labeled “positive” (perturbation of the ACR led to a significant decrease in gene expression) and 9,938 were labeled “negative” (no significant reduction in expression despite good power). We performed this analysis in correlated ACR–gene pairs as defined above.

**Topologically-associated domain (TAD) analyses**

We used previously published data of HSPC TADs (*34*). We reprocessed data using Juicer Tools 1.19.02 HiCCUPS using default settings to obtain loops and used *arrowhead* to obtain TADs. To obtain control accessibility-matched ACRs and expression-matched genes, we generated a distribution using non-TF sensitive elements with similar mean and skew to the distribution of TF-sensitive ACRs and randomly sampled an equal number of observations. By randomly sampling this distribution 100 times, we built a baseline expectation of the probability of a given TF-ACR pair to be found within a TAD. We performed this analysis for ACR–gene links split in 50kb genomic distance bins, to include similar numbers of TF-sensitive and control ACR–gene links in each bin.

**GATA1 and NFE2 ChIP-seq analyses**

We used previously published GATA1 ChIP-seq data from day 12 primary human erythroblasts (*36*), and previously published NFE2 ChIP-seq data from GYPA^+^ primary human erythroblasts (*57*). We reprocessed the data and mapped reads using BWA 0.7.15. We only kept uniquely mapped reads to the human genome using samtools view -q 1. We then called GATA1 and NFE2 peaks using MACS2. To perform metaplots, we used deeptools *computeMatrix* and *plotProfile.* To compute the profiles of *GATA1* and *NFE2* chromatin accessible TF-sensitive peaks, *computeMatrix* and *plotProfile* were run surrounding GATA1 or NFE2 peaks computed using MACS2.

**Genomic non-coding constraint of haploinsufficient variation (Gnocchi) scores**

We obtained genome-wide constraint z-scores from gnomAD 3.1 for 1kb windows and overlapped them with our functional elements (i.e., non-accessible chromatin, TF-sensitive accessible ACRs, and non-TF-sensitive ACRs) using the *GenomicRanges* function. Z-scores were downloaded from <https://gnomad.broadinstitute.org/downloads#v3-genomic-constraint>, as previously described (*39*). The genomic constraint is quantified using a 1 kb scale with a Z score obtained by comparing the observed variation to an expectation. In brief, the expected number of variants in each tiling 1 kb genomic region is predicted using a mutational model that takes into consideration local sequence context and multiple genomic features. The higher the positive Z score (i.e., fewer observed variants than expected), the higher the constraint (*39*).

**Blood cell trait credible set and TF-sensitive element overlap analysis**

We computed the number of 95% credible sets overlapping with TF-sensitive ACRs for each blood cell trait from one of the largest GWAS for blood cell traits performed to date, Chen et al. (*2*). To build control distributions, we obtained 100 random samples of the same number of non–TF–sensitive ACRs, and 100 random samples of the same number of genomic regions, with the same width as the average width of TF-sensitive ACRs.

**Heritability and LD score regression analyses**

We used data from European Ancestry from one of the largest GWAS for blood cell traits performed to date, Chen et al. (*2*). We ran stratified LD score regression (LDSC) (*63*) on our annotations alongside the baseline model for each of the fine-mapped summary statistics to compute heritability estimates and enrichments for each trait (proportion of heritability / proportion of SNPs in functional annotation). We used summary statistics and LD Scores from 1000 Genomes and European ancestry.

To define ACRs correlated with erythroid differentiation, we used Palantir with default settings on the ATAC dimension to identify sets of ACRs that monotonically increased over the course of differentiation (*64*). Briefly, Palantir aligns cells along differentiation trajectories. We aligned cells transduced with non-targeting control sgRNAs along the erythroid differentiation trajectory. Palantir models differentiation as a stochastic process in which stem cells differentiate to terminally-differentiated cells through a series of steps along a low-dimensional phenotypic manifold. Finally, we clustered all ~230,000 ACRs by their trends along this trajectory and selected trends that monotonically increased over the course of erythroid differentiation **(fig. S8C)**.

**Motif-enrichment analysis**

Analysis of overrepresented motifs in TF-sensitive ACRs correlated with erythroid differentiation compared to ACRs correlated with erythroid differentiation alone was performed using Signac *(60).* Briefly, motifs from the JASPAR 2020 database (*65*) were retrieved and added to the single cell object using the *getMatrixSet* and *AddMotifs* functions. A hypergeometric test was used (*FindMotifs* function) to test the probability of observing a motif at a given frequency by chance in TF-sensitive ACRs correlated with erythroid differentiation, compared to ACRs correlated with erythroid differentiation alone.

**Trait-enrichment score (TRS) calculation**

We classified credible sets with a 95% probability of containing the causal GWAS variant in European ancestry as overlapping either TF-sensitive or non-TF-sensitive regions (i.e., the fraction of all accessible ACRs in this experiment that were not TF-sensitive). We then used Scavenge to obtain trait-enrichment scores using variants included within credible sets overlapping TF-sensitive and non-TF-sensitive regions, as previously described (*66*). Similar enrichments were observed using only variants from the 95% credible sets directly overlapping with the aforementioned elements.

Supplementary Text

Step-by-step protocol for CROP-seq transcript PCR enrichment

Please see **fig. S11** for a graphical schematic of Perturb-multiome’s biotinylation enrichment procedure to retrieve the identity of the sgRNA.

**Procedure**

Prepare master mix for PCR1

PCR 1 reaction:

| NEBNext Q5U master mix | 15uL |
| --- | --- |
| F_CROPseq_PCR1, 25uM | 1.25uL |
| AAO272, 25uM | 1.25uL |
| Water | 11.5uL |
| 10X cDNA at 10ng/uL | 1uL |
| Total volume | 30uL |

**Note:** starting with a 30-50ng/uL concentration might increase library complexity depending on the sample.

F_CROPseq_PCR1: /5Biosg/UAUAGTGACTGGAGTTCAGACGTGTGCTCTTCCGATC**TCGATTTCTTGGCTTTATATATCTTGTG**

AAO272: CTACACGACGCTCTTCCGAT*C*T

**Note:**  KAPA HiFi Uracil+ Ready Mix works equally well - adjust annealing temperature accordingly.

**Note:** cDNA input amount might require optimization depending on the expression levels in the cell type.

Run PCR1:

98C for 30s

Repeat 8 cycles:

- - 1. 98C for 15s
    2. 69C for 15s
    3. 72C for 20s

72C for 120s

Hold at 4C

**Note:** Number of PCR1 cycles might require optimization depending on the expression levels in the cell type.

Perform SPRI purification using 1X beads by adding 30 uL of SPRIselect beads, thoroughly mixing with a pipette, and allowing it to incubate for a duration of 5 minutes. Afterwards, position it on position HIGH of the magnet and dispose of the supernatant. Follow this by washing twice using ethanol, centrifuge to concentrate, and eliminate any residual ethanol. Elute in 24µL of buffer EB. **The protocol can be stopped here at -20C.**

**Perform biotin pull-down using Dynabeads MyOne streptavidin beads as per invitrogen protocol, reproduced below:**

Prep **2X BW buffer**; see recipe from the manufacturer:

10 mM Tris-HCl (pH 7.5)

1 mM EDTA

2 M NaCl

- 1. Prepare Dynabeads MyOne streptavidin beads C1. Perform this cleanup in a single 1.5mL eppendorf for all your reactions (multiply the volumes below by your number of reactions).
     1. Prep **2X BW buffer** with the protocol above
     2. Add 12uL of Dynabeads.
     3. Add 50uL of **1X BW buffer**. Pipette mix.
     4. Place on magnet (high position) and discard supernatant
     5. Resuspend with 50uL 1x BW.
     6. Repeat steps 4 and 5 3 more times for a total of 4 washes
  2. Add 24ul **2x BW buffer** to washed Dynabeads. Distribute into as many PCR strip wells as reactions that you have. Then, add to each the 24uL of PCR product.
  3. Incubate for **15 mins** at **RT** on **hula mixer.**
  4. Place on magnet (high), remove supernatant. Wash beads three times with 50µl **1X BW buffer** (note: pipette mix each wash to resuspend the beads). **Keep the beads.**
  5. Resuspend in **15uL trisEDTA (TE)** + **0.5uL NEB USER enzyme**. Incubate for **30 minutes** (incubation can safely be reduced from 2 hours) at 37degC on ***hula mixer.***
  6. Place on magnet (low), **transfer the supernatant** to a clean PCR tube.
  7. Perform **1X SPRI cleanup** (add 15.5 uL of SPRIselect beads, pipette mix and incubate for 5 minutes, magnet and discard supernatant, wash 2x with 200µl 80% ethanol, spin down and remove any remaining ethanol). Elute in 15uL Elution Buffer (Qiagen) or H2O, incubate 2min at RT, place on magnet low and transfer the supernatant in another PCR strip.

Perform **PCR2, 27 cycles. This should be optimized for your cell type/experiment/MOI using qPCR: you can use 2.5uL of the prior elution for qPCR. We find 27-28 cycles is optimal for HSPCs.**

| NEB Q5 MASTER MIX (NEB, M0492L) | 25uL |
| --- | --- |
| Universal Illumina adapter P5, **100uM**  AATGATACGGCGACCACCGAGATCTACACTCTTTCCCTACACGACGCTC | 0.5uL |
| Universal Illumina adapter P7, **100uM** CAAGCAGAAGACGGCATACGAGATXXXXXXXXGTGACTGGAGTTCAGACGTGTGCTCTTCCGATCT  => Add a different index for each reaction | 0.5uL |
| Clean PCR1 product | 12.5uL |
| Water | 11.5uL |
| Total reaction | 50uL |

Run PCR2:

98C for 30s

Repeat 27-28 cycles:

- - 1. 98C for 15s
    2. 69C for 15s
    3. 72C for 20s

72C for 120s

Hold at 4C

Perform 1X SPRI cleanup (**50uL of beads**) and elute in 30uL of EB buffer.

Run 1µl of each PCR reaction on a gel to make sure it amplified

Pool 2µl of each PCR for the final library pool, then dilute 1:10 and Run BioA.

**Illumina Sequencing**

28 cycles R1 (cell barcode and UMI), 8 cycles I1 (sample index), 20 cycles R2 (sgRNA sequence, use Custom_sequencing_primer_R2_CROPseq).

| Custom_sequencing_primer_R2_CROPseq: CGATTTCTTGGCTTTATATATCTTGTGGAAAGGACGAAACACCG |
| --- |

**Selection of Transcription Factors (TFs)**

The rationale for determining the transcription factors to target relies on existing literature. Some TFs have been shown to be sufficient to initiate the direct transversion of human fibroblasts to erythroid differentiation (GATA1, TAL1, LMO2, cMYC, KLF1, MYB) *(67),* and are *de facto* master TFs of erythropoiesis. This restricted set of factors, although necessary to shape the erythroid commitment, does not illustrate the complexity of erythropoiesis in human cells. Other transcription factors play important roles in terminal maturation or in the control of very specific mechanisms. This is the case of BCL11A, which initiates the switch from fetal to adult hemoglobin [*(*](https://paperpile.com/c/OfZ7hU/zAtH)*23*[*)*](https://paperpile.com/c/OfZ7hU/zAtH) and likely plays minor developmental roles in the red blood cell lineage [*(*](https://paperpile.com/c/OfZ7hU/YZhp)*68*[*)*](https://paperpile.com/c/OfZ7hU/YZhp)*.*

LDB1, which forms part of the big TAL1 complexes with LMO2 and GATA1, was also an interesting factor to target, although there is no evidence for direct DNA binding [*(*](https://paperpile.com/c/OfZ7hU/ipw3)*69*[*)*](https://paperpile.com/c/OfZ7hU/ipw3)*.*

To account for the diversity of TFs involved in hematopoiesis, we sought to include targets likely to impact erythroid differentiation at later stages, like ATF4, IRF1, or IRF9, but also early TFs regulating the commitment to erythroid and non-erythroid myeloid fates, like SPI1 (also known as PU.1), GATA2, or RUNX1. To get a better sense of the TF activity at various stages of early myelopoiesis, early erythroid commitment, and late erythroid maturation, we relied on the identification of the most variable TF binding-motifs expressed throughout in-vitro erythroid maturation from earlier work [*(*](https://paperpile.com/c/OfZ7hU/lXSo)*21)*.

**Additional details about the Mixscale perturbation score**

The Mixscale framework comprises the steps below, which we perform for the scRNA-seq and scATAC-seq matrices independently **(fig. S12)**. Full details can be found in the Mixscale preprint [(](https://paperpile.com/c/OfZ7hU/7lTLN)*27*[)](https://paperpile.com/c/OfZ7hU/7lTLN).

(1) For each cell with a TF-targeting sgRNA, its *k* nearest neighbors with a control sgRNA are identified and the averaged mRNA expression profile of the control neighbor cells is subtracted from the mRNA expression profile of the target cell. As a result, shared variation is mitigated and perturbation-specific effects are reinforced **(fig. S13)**.

(2) Differential gene expression or chromatin accessibility analysis is conducted between cells with TF-targeting sgRNAs and cells with control sgRNAs.

(3) Using the corrected expression values from step 1, a perturbation vector with length equal to the number of identified differentially expressed genes or accessible chromatin regions is defined.

(4) The average difference in perturbation vectors between targeted and control cells is computed. The perturbation vector from step 3 is projected onto this difference vector to obtain the perturbation score for each cell. This score is a representation of the expression or chromatin accessibility shift of a given cell from control cells due to the perturbation (the higher the perturbation score, the greater the perturbation response).

(5) The perturbation scores in TF-targeting cells are then standardized using the mean and standard deviation of the scores in control cells.

(6) Weighted multivariate regression to identify differential gene expression or chromatin accessibility analysis is performed using the standardized scores as input. As such, the perturbation score gives perturbed cells bigger weight in the discovery of TF-sensitive genes and ACRs. Sequencing depth is added as a covariate in the regression. To avoid circularity in this strategy, a leave-one-out approach is used, in which the gene or ACR predicted in the regression is taken out from the initial perturbation score computation.

We assessed the calibration of our association analyses by applying them to control sgRNAs (non-targeting and AAVS1 sgRNAs) **(fig. S14)**.

#
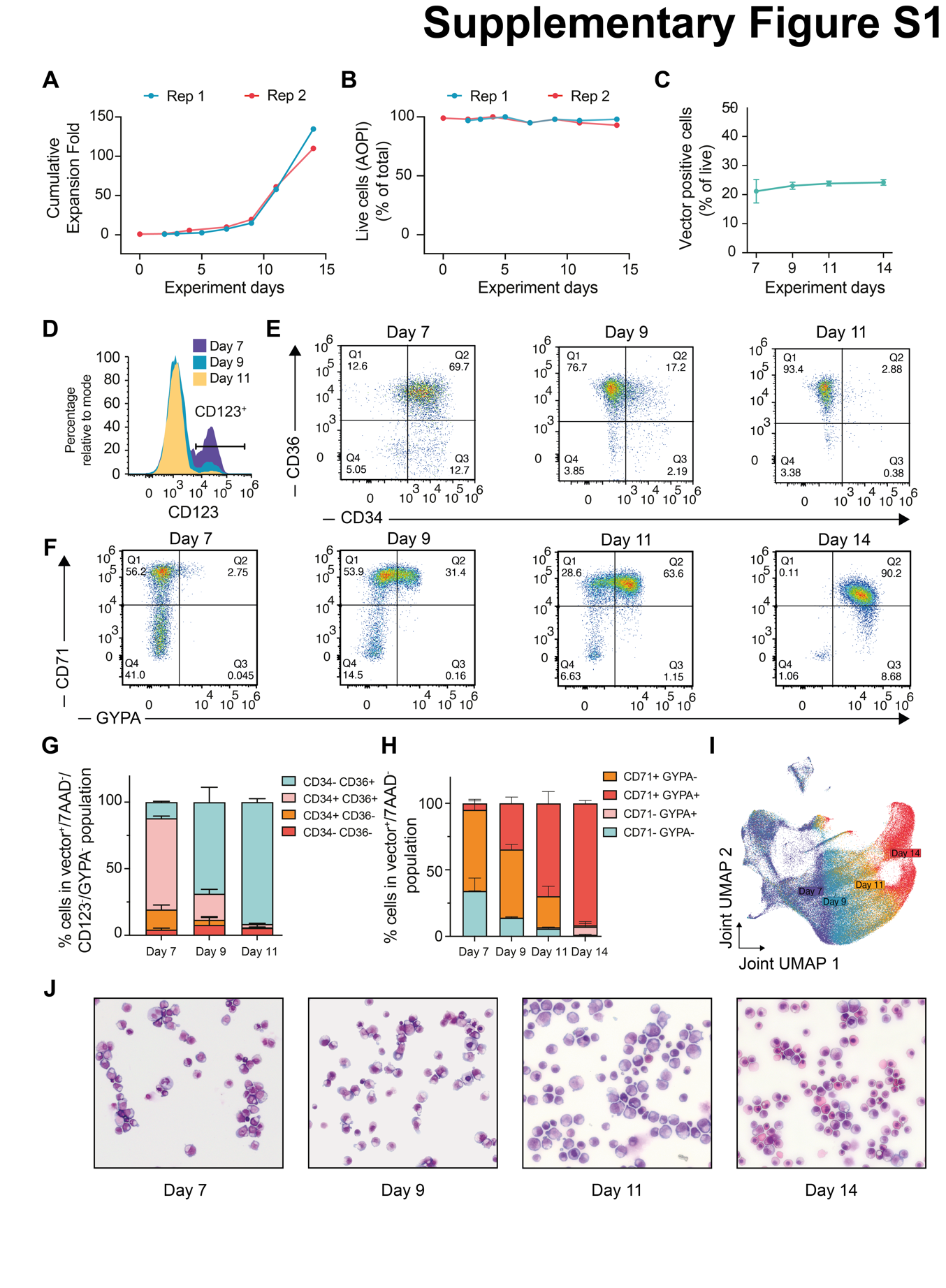


| **Supplementary Figure 1. Characterization of erythroid differentiation in cells subjected to Perturb-multiome.** |
| --- |

(A) Cell growth curves of two experimental replicates over the course of the experiment described in fig. 1.

(B) Cell viability curves of two experimental replicates over the course of the experiment described in fig. 1.

(C) Percentage of differentiating hematopoietic cells expressing the lentiviral vector over each of the experimental time points, following transduction at low multiplicity of infection. Cells were subsequently enriched using FACS to obtain a pure population (>99%) of transduced cells prior to single-cell profiling.

(D) Histograms of CD123 (IL3RA) protein expression over the course of experimental time points, showing the progressive enrichment of erythroid cells.

(E) Scatter plots of CD34 and CD36 protein expression over the course of experimental time points, showing the progression through erythroid progenitor stages.

(F) Scatter plots of GYPA and CD71 protein expression over the course of experimental time points, showing the progressive maturation of erythroid precursors.

(G) Bar plots for two biological replicates of coordinated changes in CD34 and CD36 protein expression across time points. Mean and standard deviation are displayed.

(H) Bar plots for two biological replicates of coordinated changes in GYPA and CD71 protein expression across time points. Mean and standard deviation are displayed.

(I) UMAP reduction using a weighted-nearest neighbor graph (*56*) to integrate RNA and chromatin accessibility peak information from the same single cells. Cells are colored by the experimental timepoint of profiling.

(J) Representative microscopy images of sorted cells at each of the experimental time point, used profiled with Perturb-multiome. Cells progress from immature stages with large nucleus and apparent nucleolus (Day 7) to early erythroblasts with large basophilic cytoplasm (Day 9), then decreasing in size and compacting chromatin (Day 11), and ultimately cytoplasm turning polychromatophilic and then acidophilic before enucleation (Day 14) at terminal stages.


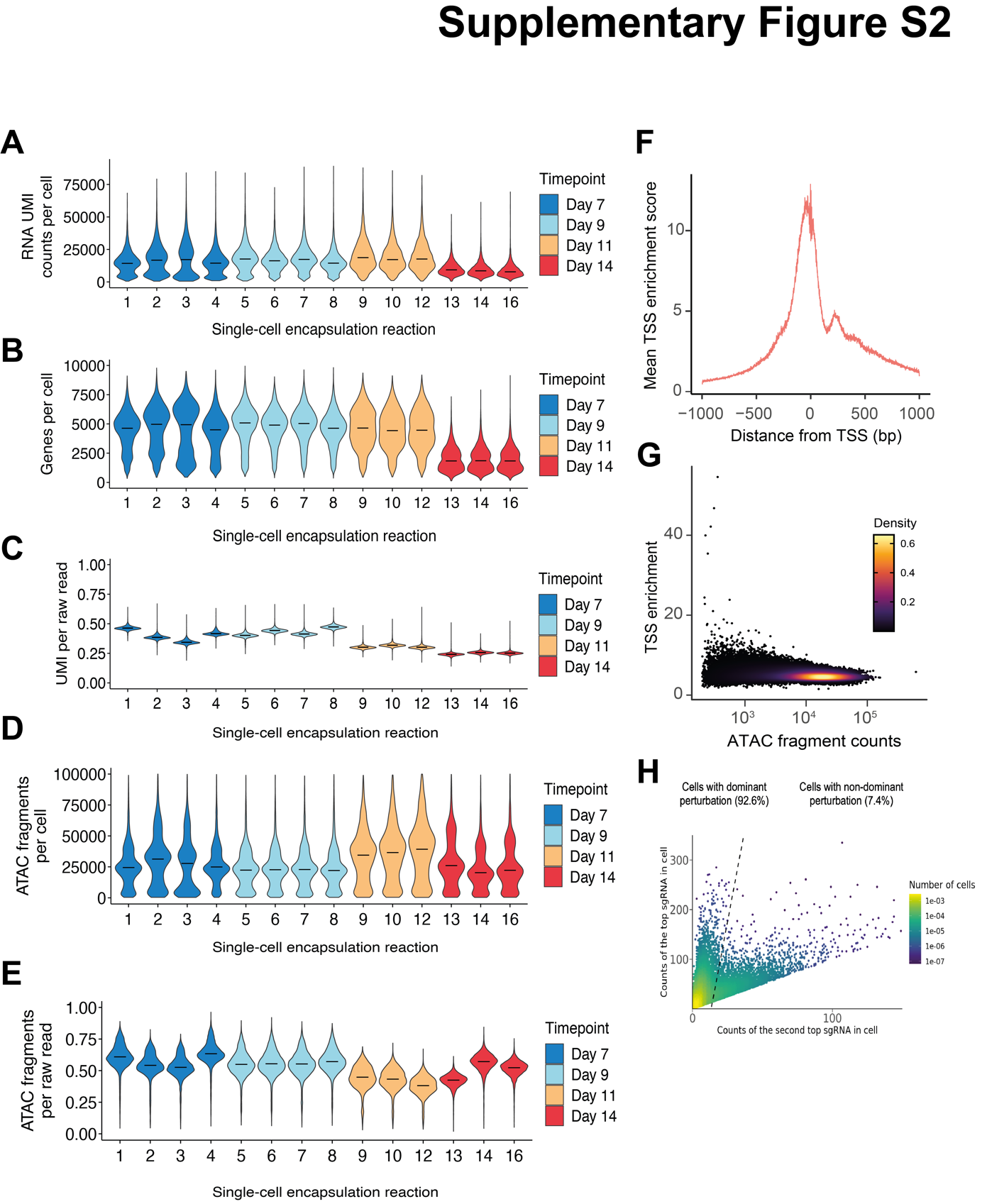


| **Supplementary Figure 2. Quality metrics of Perturb-multiome.**  (A) Violin plots of the RNA UMI counts per single cell, grouped by single-cell encapsulation reaction and colored by experimental time point. Horizontal black lines represent the median for each group. |
| --- |

(B) Violin plots of the genes detected per single cell, grouped by single-cell encapsulation reaction and colored by experimental time point. Horizontal black lines represent the median for each group.

(C) Violin plots of the ratio between the number of UMI per cell and the number of gene expression raw sequencing reads per cell, grouped by single-cell encapsulation reaction and colored by the time point of experimental collection. Horizontal black lines represent the median for each group.

(D) Violin plots of the ATAC fragments per single cell, grouped by single-cell encapsulation reaction and colored by experimental time point. Horizontal black lines represent the median for each group.

(E) Violin plots of the ratio between the number of ATAC fragments per cell and the number of ATAC raw sequencing reads per cell, grouped by single-cell encapsulation reaction and colored by the time point of experimental collection. Horizontal black lines represent the median for each group.

(F) Mean transcription start site (TSS) enrichment score as a function of the distance to the TSS for all single cells in the experiment.

(G) Density plot of the per-cell TSS enrichment score as a function of the number of ATAC fragment counts in each cell.

(H) Density plot of the CROP-seq UMI counts of the top sgRNA in each cell as a function of the CROP-seq UMI counts of the second sgRNA with most counts in each cell.


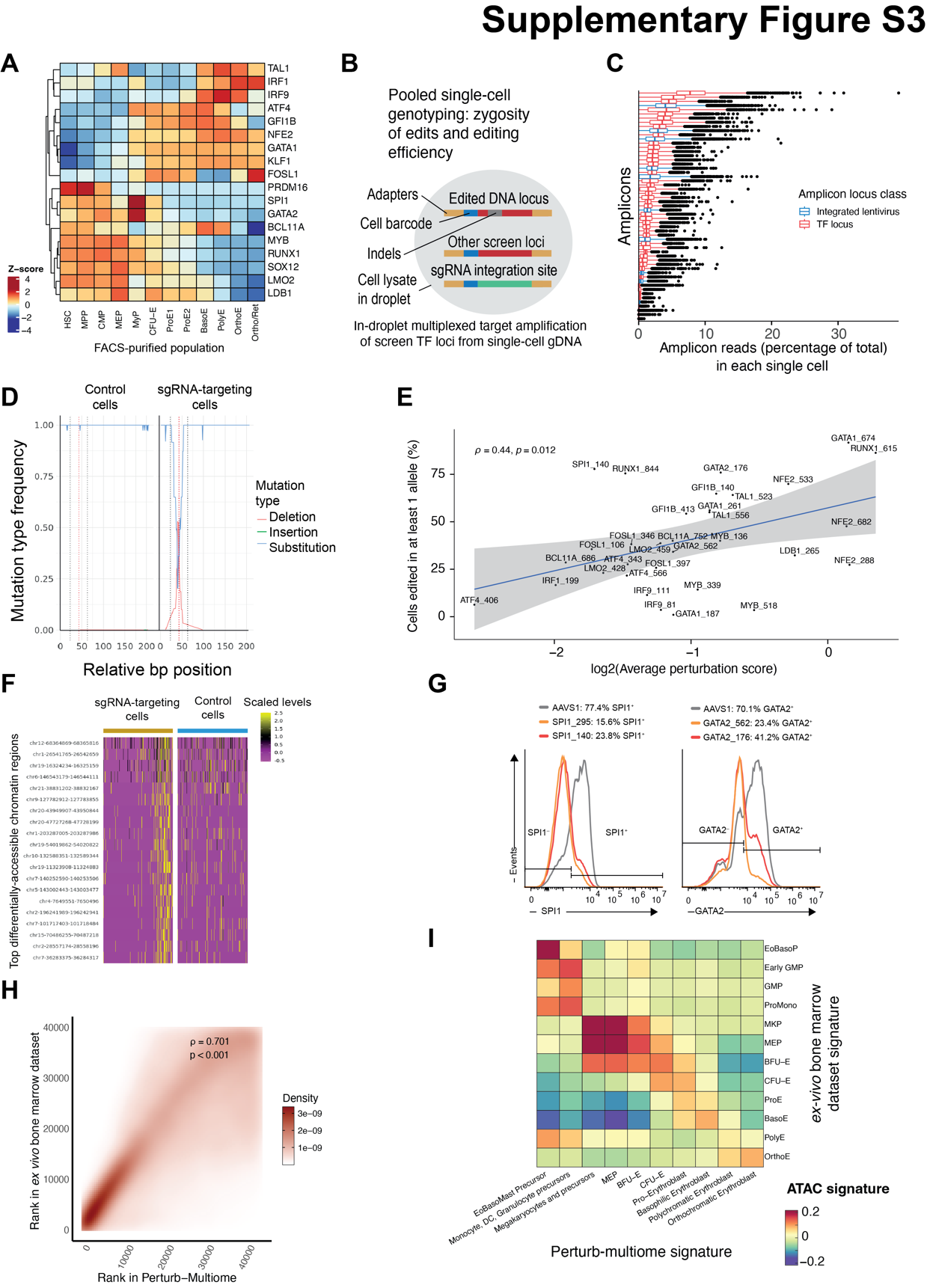


**Supplementary Figure 3.** **Assessment of transcription factor perturbation efficiencies.**

(A) Heatmap of the Z-scored bulk RNA expression levels for some of the transcription factors (TFs) targeted in the screen in FACS-purified populations, using data from (*21*). Hierarchical clustering, represented on the dendrogram, was performed across TFs. *SNAI2,* a TF also perturbed in our experiment, was not reported in this dataset.

(B) Schematic of pooled single-cell screen genotyping for screen editing efficiency assessment using multiplexed single-cell PCR.

(C) Box plots of the per cell percentages represented by each genotyping, colored by amplicon locus class.

(D) Pseudo-bulk single-cell insertion, deletion, and substitution frequencies at one of the targeted RUNX1 loci, shown for control cells and cells with targeting sgRNAs. Mutational frequencies are a weighted frequency of the times that a given type of mutation was seen across reads for one cell barcode, which was then averaged across cells. Black dotted lines represent the boundaries of the sgRNA and the red dotted line represents the predicted cut site.

(E) Scatter plot of the percentage of single-cells with at least one allele edited for each sgRNA from the pooled single-cell genotyping experiment and the average log_2_(average perturbation score) computed on RNA from the Perturb-multiome experiment. The Spearman correlation coefficient and a regression line with confidence intervals are shown.

(F) Heatmaps of the scaled levels of the top differentially-accessible chromatin peaks between GATA1 sgRNA-targeting and control cells. Within each heatmap, each column represents a sample of single cells, ordered by the perturbation score.

(G) Left: histograms of the flow cytometry measurements of intracellular TF SPI1 protein levels for HSPCs edited with AAVS1 control, SPI1_295, or SPI1_140 sgRNA. Right: histograms of the flow cytometry measurements of intracellular TF GATA2 protein levels for HSPCs edited with AAVS1 control, GATA2_562, or GATA2_176 sgRNA.

(H) Density plot of the ranks of the top 25% ACRs in an *ex vivo* bone marrow dataset in our Perturb-multiome *in vitro* dataset (x-axis) and the *ex vivo* dataset (y-axis). The Spearman correlation coefficient and the p-value are displayed. 93% of the top 25% ACRs are shared between both datasets.

(I) Heatmap of ACR single-cell signature scores calculated using marker ACRs identified from the *ex vivo* bone marrow dataset, averaged per cell state for Perturb-multiome (columns) and the *ex vivo* bone marrow dataset (rows). Column labels: EoBasoMast = Eosinophils, Basophils, Mastocyte; DC = Dendritic Cells; MEP = Megakaryocyte and Erythrocyte Precursors; BFU-E = Burst Forming Unit-Erythroid; CFU-E = Colony Forming Unit-Erythroid. Row labels: EoBasoP = Eosinophil and Basophil Progenitors; GMP = Granulo-Monocytic Precursors; ProMono = Pro-monocytes; MKP = Megakaryocytic Precursors; MEP = Megakaryocyte and Erythrocyte Precursors; BFU-E = Burst Forming Unit-Erythroid; CFU-E = Colony Forming Unit-Erythroid; ProE = Pro-Erythroblasts; Baso-E = Basophilic Erythroblasts; PolyE = Polychromatophilic Erythroblasts; OrthoE = Orthochromatophilic Erythroblasts.


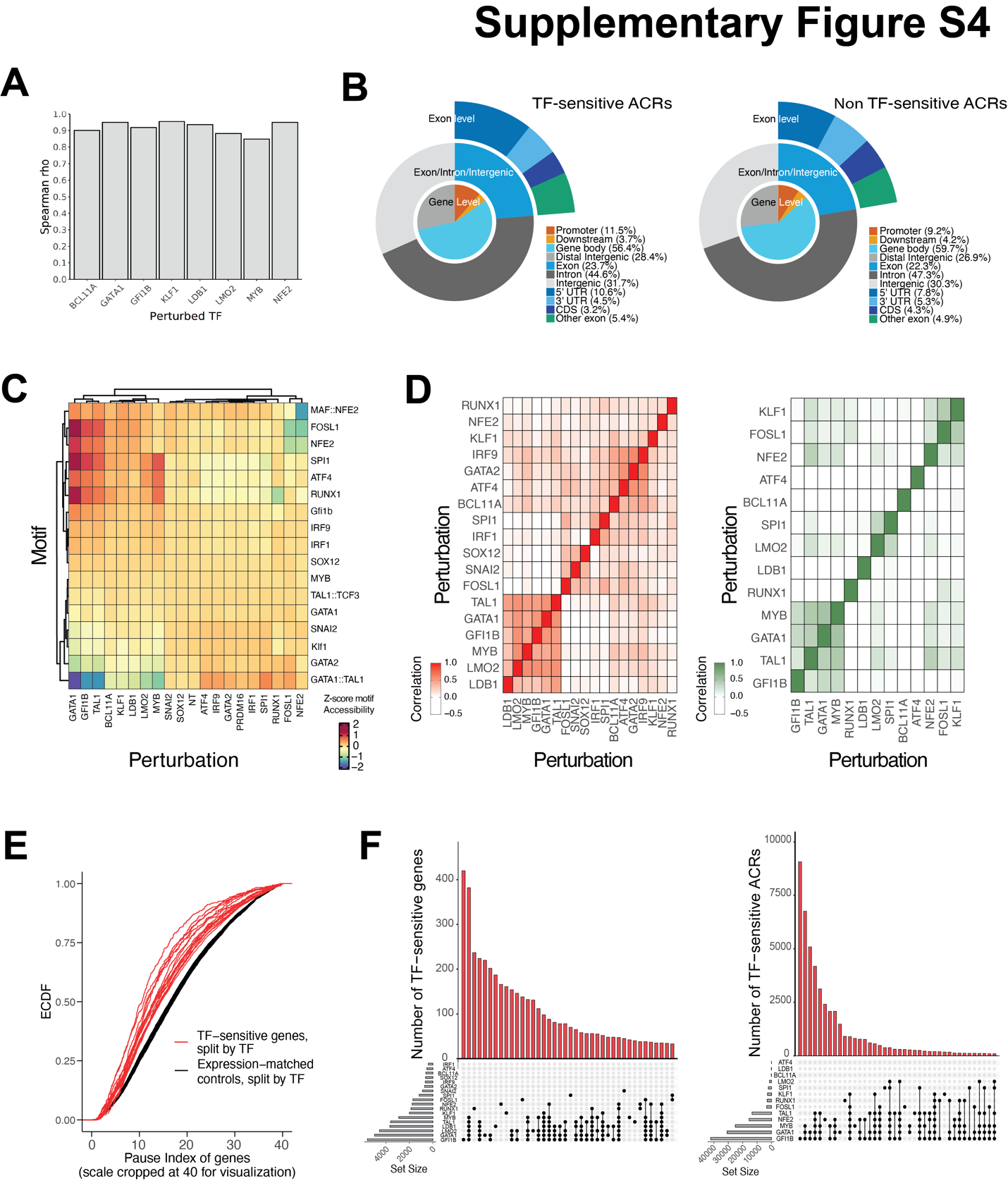


**Supplementary Figure 4. Properties of TF-sensitive genes and cis-regulatory elements.**

(A) Spearman correlation coefficients of the gene expression model weights (i.e. the degree of perturbation of each gene) in two biological replicates that each used different HSPC donors and sgRNAs targeting different locations of the TF coding region. Data shown for TFs perturbed in both replicates.

(B) Distribution of genomic features in TF-sensitive (left panel) and non-TF-sensitive ACRs (right panel) by genomic regions.

(C) Heatmap of TF motif accessibility (rows) for each perturbed TF in our dataset (columns), calculated in single cells and averaged per perturbation.

(D) Heatmaps of pairwise correlation (Spearman) for the proportion of shared TF-sensitive genes (left panel, red scale) and shared ACRs (right panel, green scale). Correlation coefficient was calculated only for perturbations showing significant TF-sensitive ACRs.

(E) Cumulative distribution plot of the pause index calculated with PRO-seq of TF-sensitive genes for each TF (red) and control expression matched genes for each (black).

(F) Left, upset plot of the overlap between the TF-sensitive genes arising from the perturbation of each transcription factor. Right, upset plot of the overlap between the TF-sensitive ACRs arising from the perturbation of each transcription factor.

**
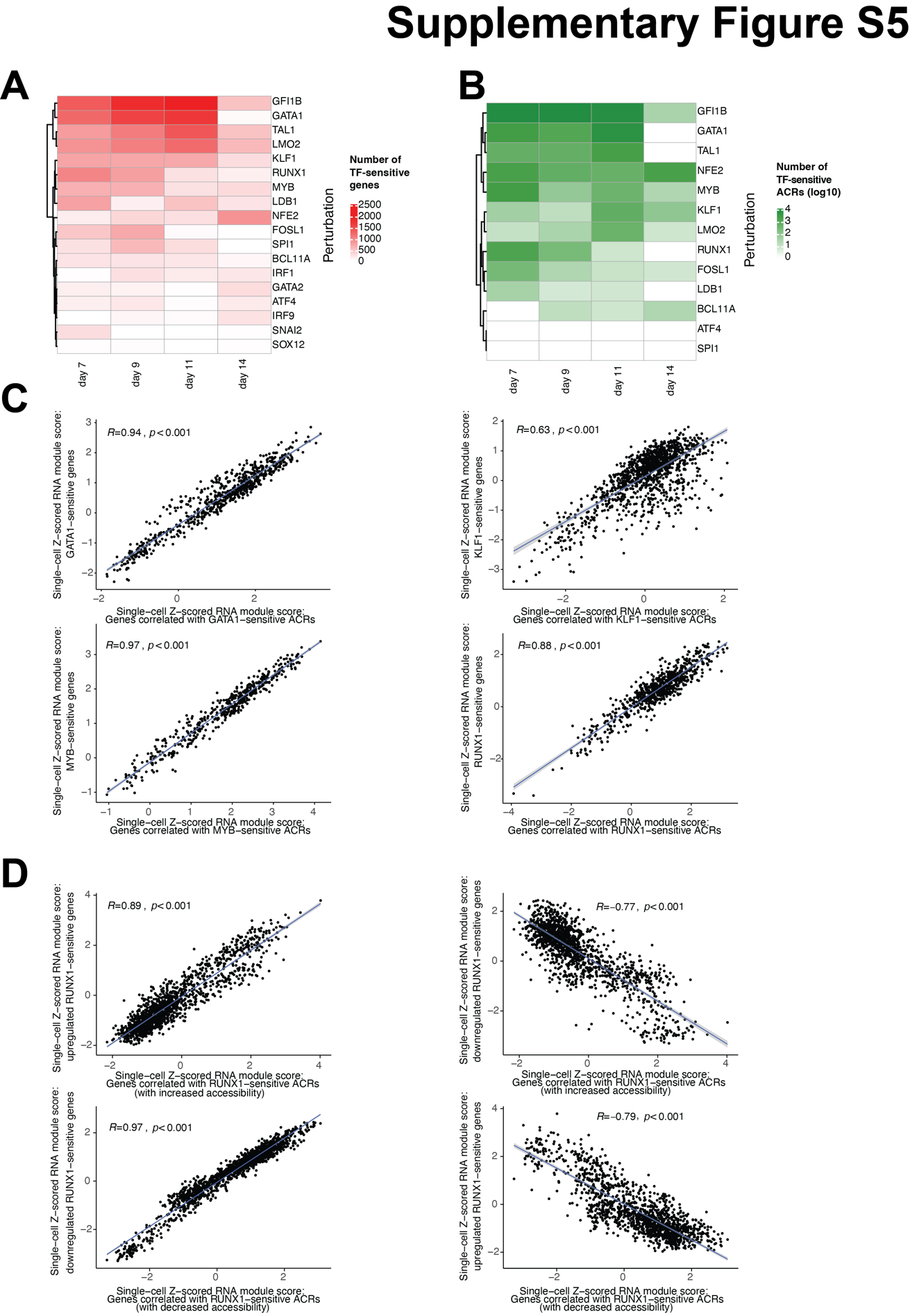
**

**Supplementary Figure 5.**  **Characterization of TF-sensitive genes and ACRs by timepoint and correlations between TF-sensitive genes and genes correlated with TF-sensitive ACRs**

(A) Heatmap of the number of TF-sensitive genes, when running analyses by timepoint and TF perturbation. Only perturbations with detected TF-sensitive genes are represented.

(B) Heatmap of the number of TF-sensitive ACRs, when running analyses by timepoint and TF perturbation (log_10_ color scale). Only perturbations with detected TF-sensitive ACRs are represented.

(C) Scatter plot of the single-cell Z-scored RNA module score computed using MYB, GATA1, KLF1 or RUNX1-sensitive genes (defined as in Figure 2B) and the single-cell Z-score RNA module score computing using genes correlated with MYB, GATA1, KLF1 or RUNX1-sensitive ACRs (defined as in panel Figure 2C, Materials and Methods), for cells with a Z-scored perturbation score greater than 1. The Spearman correlation coefficient ρ is shown, as well as a regression line with confidence intervals.

(D) Scatter plot of the single-cell Z-scored RNA module score computed using RUNX1-sensitive genes and the single-cell Z-score RNA module score computing using genes correlated with RUNX1-sensitive ACRs, for cells with an absolute Z-scored perturbation score greater than 1. The Spearman correlation coefficient ρ is shown, as well as a regression line with confidence intervals. Top left, upregulated RUNX1-sensitive genes vs. genes correlated with opening RUNX1-sensitive ACRs. Top right, downregulated RUNX1-sensitive genes vs. genes correlated with opening RUNX1-sensitive ACRs. Bottom left, downregulated RUNX1-sensitive genes vs. genes correlated with closing RUNX1-sensitive ACRs. Bottom right, upregulated RUNX1-sensitive genes vs. genes correlated with closing RUNX1-sensitive ACRs.


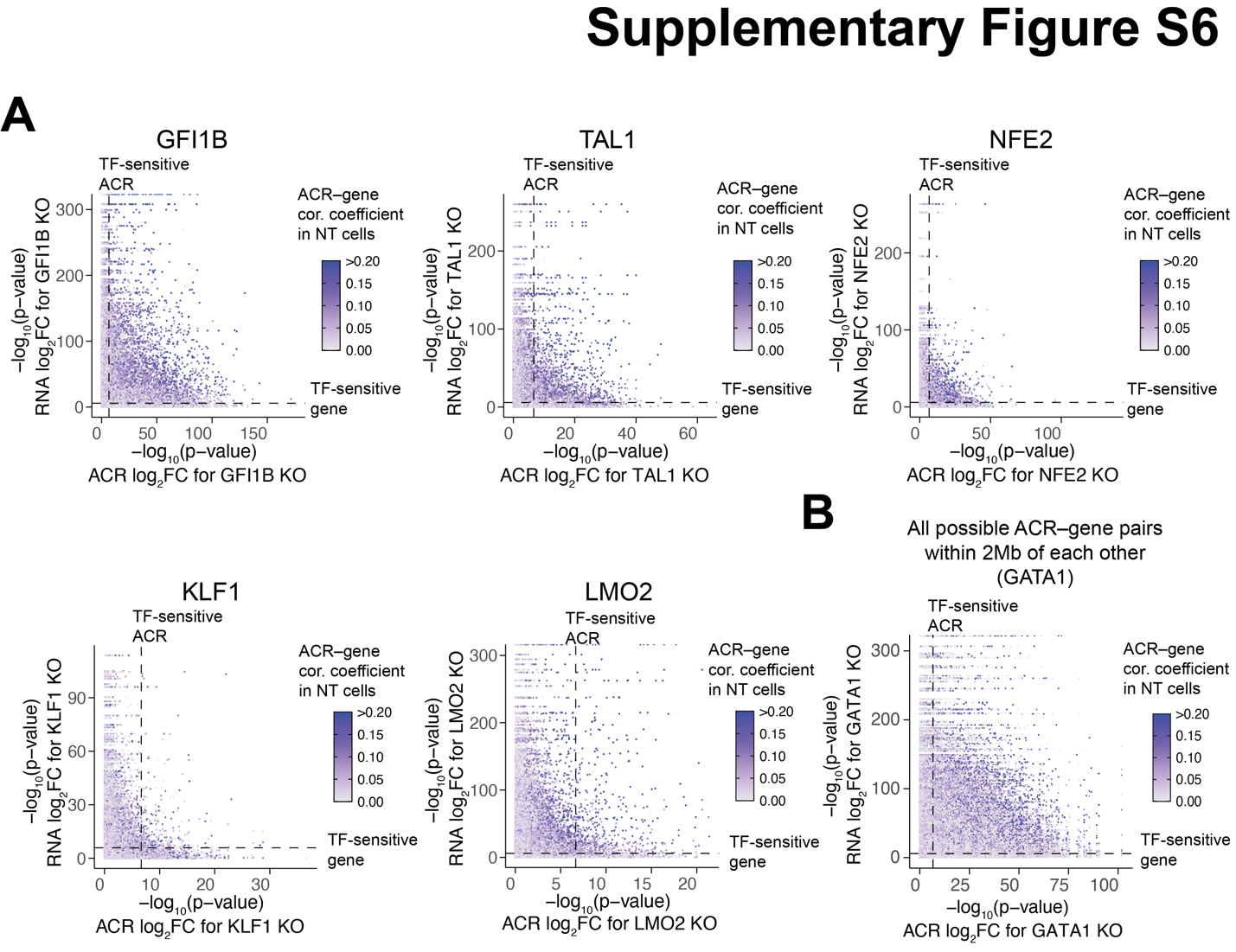


**Supplementary Figure 6. Correlation between TF-sensitive genes and TF-sensitive ACRs.**

(A) Scatter plots of the ACR and gene –log_10_(p-value) for TF-targeting sgRNAs, respectively, for all possible ACR–gene pairs within 50kb of each other. Plotted are erythroid TFs with more than 250 TF-sensitive ACR-gene pairs within 50kb from one another. Dots are colored by the ACR–gene correlation coefficient computed in cells with control NT sgRNAs (Materials and Methods). The vertical and horizontal dashed lines represent the adjusted p-value cutoffs for TF-sensitivity of ACRs and genes, respectively.

(B) Same as Figure 3B but with a 2Mb distance window.


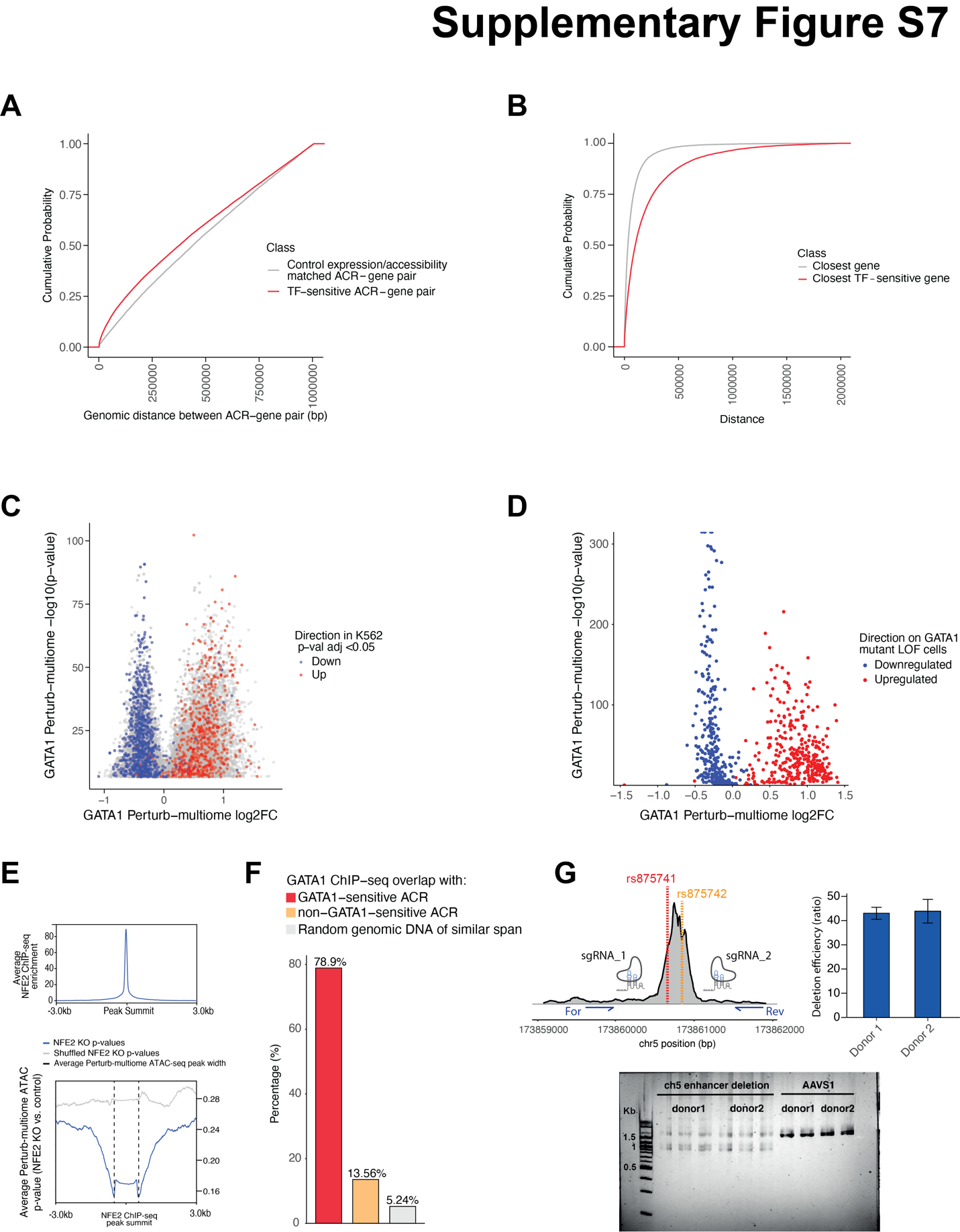


**Supplementary Figure 7. Validation of ACR and gene TF-sensitivity and correlations.**

(A) Cumulative distribution of the genomic distance (bp) between TF-sensitive ACR-gene pairs or matched expression/accessibility controls.

(B) Cumulative distribution of the genomic distance (bp) of TF-sensitive ACRs to the closest gene or to the closest TF-sensitive gene.

(C) Volcano plot displaying the -log_10_(p-value) and log_2_FC of differentially accessible ACRs from Perturb-multiome for GATA1-perturbed cells. The direction of the log_2_FC in ACRs with adjusted p-value <0.05 from GATA1-perturbed cells in the K562 dataset is used to color common ACRs in both datasets (in red for positive log_2_FC and in blue for negative log_2_FC).

(D) Volcano plot showing the log_2_FC and p-values of Perturb-multiome for GATA1-sensitive genes, which are colored by the direction of the effect for bulk RNA-seq in primary human hematopoietic cells with a GATA1 Loss-Of-Function (LOF) mutation compared to those transduced with Wild-Type (WT) GATA1.

(E) Top: profile plot of independent NFE2 ChIP-seq enrichment surrounding called ChIP-seq peaks from GYPA^+^ human erythroid precursors (*57*). Bottom: profile plot of Perturb-multiome p-values for the log_2_FC of accessibility for peaks in cells with NFE2-targeting sgRNAs compared to control, centered on NFE2 ChIP-seq peaks as defined in the top panel.

(F) Percentage of overlap from GATA1 ChIP-seq with GATA1 sensitive ACRs, a comparable number of non-GATA1-sensitive ACRs, or a comparable number of random genomic regions of similar span.

(G) Top left, schematic of the location of the two sgRNA targeting the GATA1-perturbation-sensitive, CPEB4 correlated ACR (in hg38 coordinates). The two vertical lines highlight two variants with pleotropic associations to blood cell traits. Bottom, PCR products for primers flanking the CPEB4 ACR, shown for edited cells and AAVS1-edited controls, respectively. Data shown for two donors. Top right, deletion efficiency ratio calculated from the area-under-the-curve using Agilent bioanalyzer on the PCR products from the CPEB4 enhancer deletion.


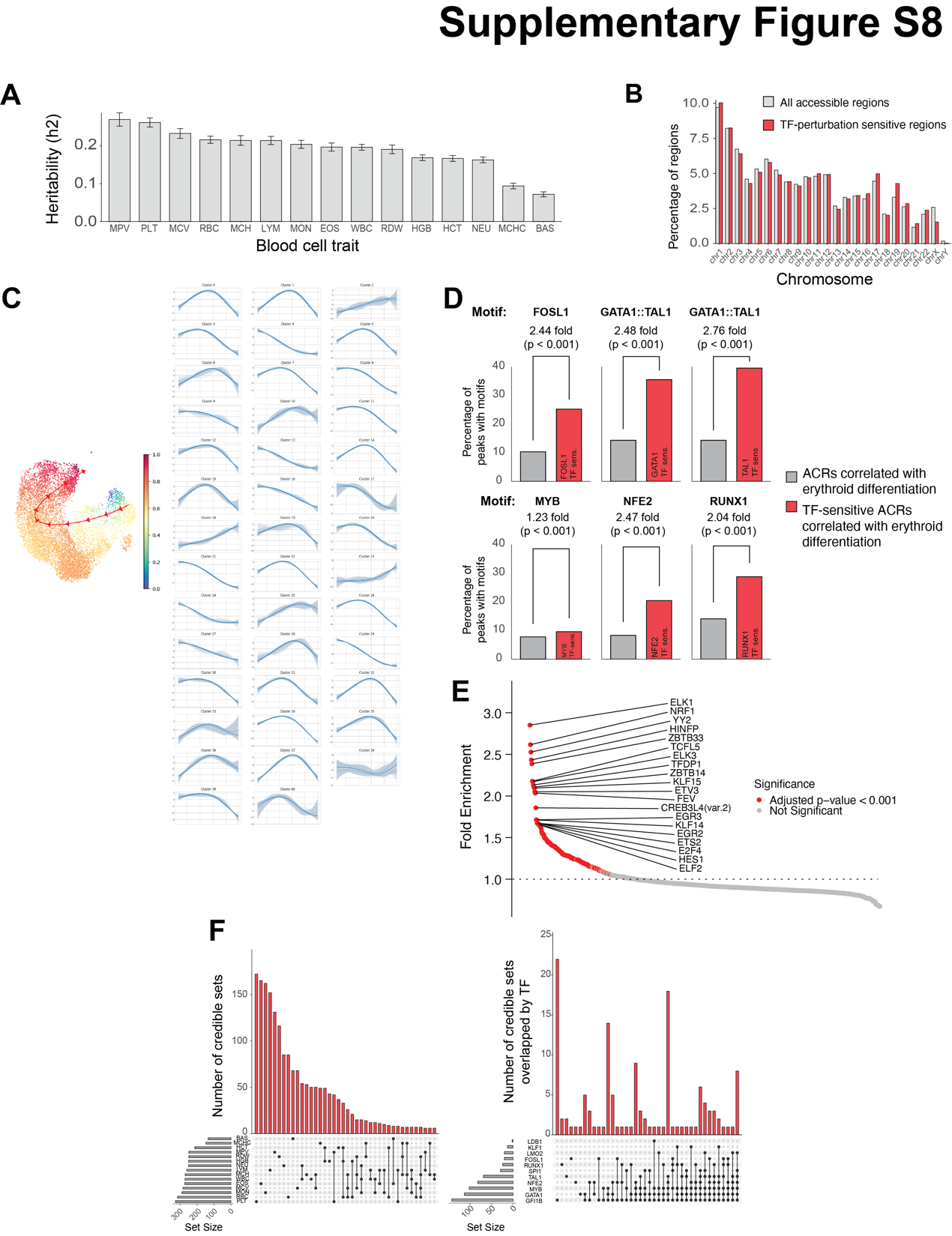


**Supplementary Figure 8. Characterization trait-relevant variant enrichment in accessible chromatin regions with Perturb-multiome.**

(A) Fraction of heritability explained by genetic variation for blood cell traits **(Materials and Methods).**

(B) Percentage of all accessible regions or TF-sensitive regions that are present in each chromosome.

(C) Left, ATAC UMAP of Perturb-multiome used as input for Palantir to identify ACRs correlated with erythroid differentiation, colored by pseudotime **(Materials and Methods).** The red line shows the trajectory relative to which the dynamics of accessibility changes were identified. Right, clusters of ACR dynamic behaviors over the course of the aforementioned trajectory. Those that increased monotonically over the course of differentiation were defined as correlated with erythroid differentiation **(Materials and Methods).**

(D) Barplots of the enrichment in TF-binding motifs between TF-sensitive ACRs correlated with erythroid differentiation and ACRs correlated with erythroid differentiation alone, regardless of their TF-sensitivity status.

(E) Enrichment of transcription factor binding motifs in non-TF-sensitive accessible chromatin regions correlated with erythroid differentiation. Dots colored in red represent transcription factor (TF) binding motifs whose enrichment is significant after multiple hypothesis testing correction.

(F) Left, upset plot of the overlap between the credible sets of each blood cell trait. Right, upset plot of the overlap between the credible sets overlapped by each transcription factor.

Blood cell traits are referred as follows in the figure: BAS = basophil count, EOS = eosinophil count, HCT = hematocrit, HGB = hemoglobin, LYM = lymphocyte count, MCH = mean corpuscular hemoglobin, MCHC = mean corpuscular hemoglobin concentration, MCV = mean corpuscular volume, MON = monocyte count, NEU = neutrophil count, PLT = platelet count, RBC = red blood cell count, RDW = red cell distribution width, WBC = white blood cell count. BAS, EOS, MON, NEU, WBC are non-erythroid myeloid traits; LYM is a lymphoid trait; PLT is a megakaryocytic trait; HCT, HGB, MCH, MCHC, MCV, RBC, RDW are erythroid traits.

**
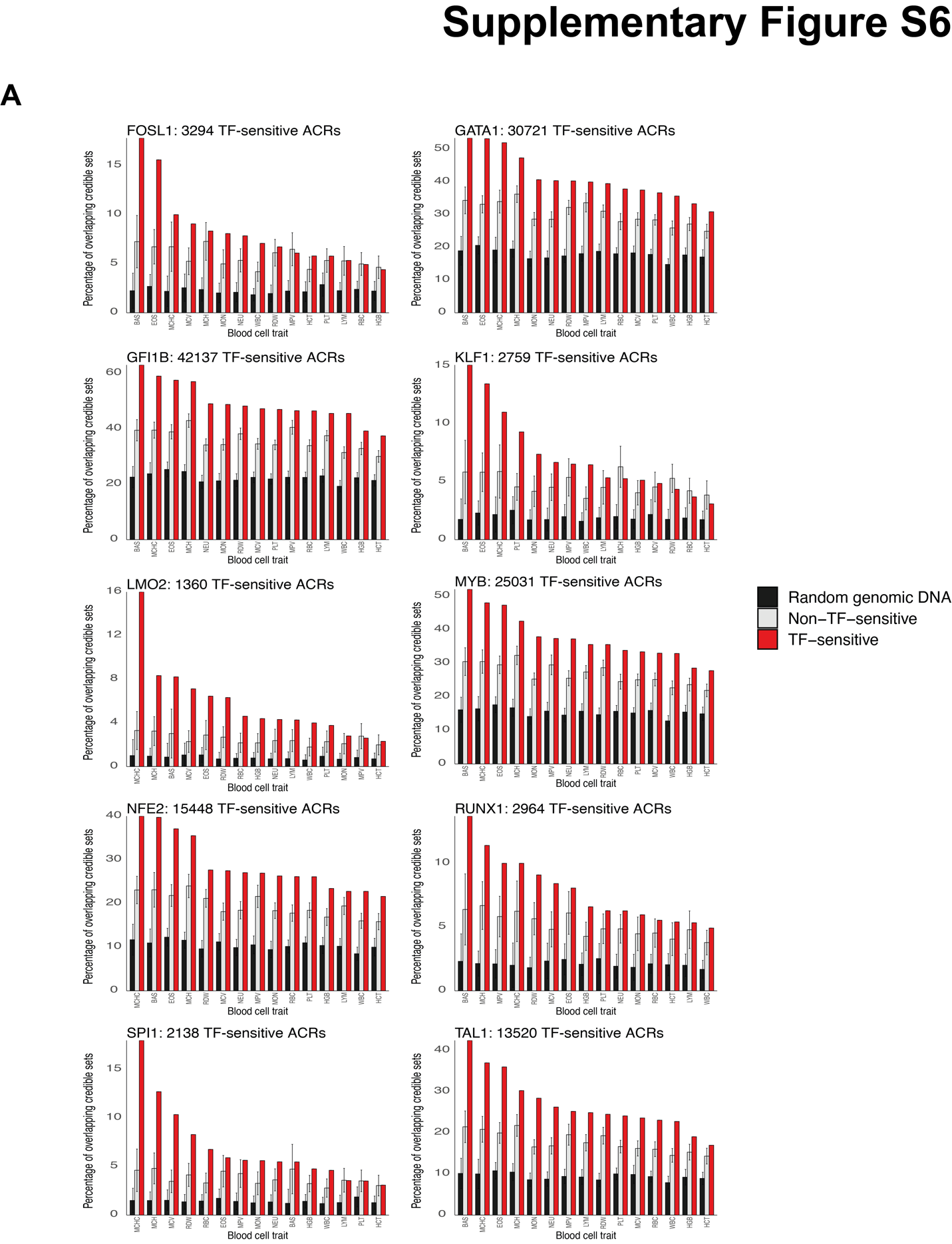
**

**Supplementary Figure 9.**  **Overlap of blood-cell trait credible sets with TF-sensitive ACRs by TF.**

Barplots for the percentage of 95% credible sets overlapping TF-sensitive ACRs, for each blood cell trait and TF, for TFs with more than 250 TF-sensitive ACRs. Error bars represent the standard deviation of 100 sampling events of non-TF-sensitive accessible ACRs (“non-TF-sensitive”) or of any genomic region (“random genomic DNA”) **(Materials and Methods).**

Blood cell traits are referred as follows in the figure: BAS = basophil count, EOS = eosinophil count, HCT = hematocrit, HGB = hemoglobin, LYM = lymphocyte count, MCH = mean corpuscular hemoglobin, MCHC = mean corpuscular hemoglobin concentration, MCV = mean corpuscular volume, MON = monocyte count, NEU = neutrophil count, PLT = platelet count, RBC = red blood cell count, RDW = red cell distribution width, WBC = white blood cell count. BAS, EOS, MON, NEU, WBC are non-erythroid myeloid traits; LYM is a lymphoid trait; PLT is a megakaryocytic trait; HCT, HGB, MCH, MCHC, MCV, RBC, RDW are erythroid traits.


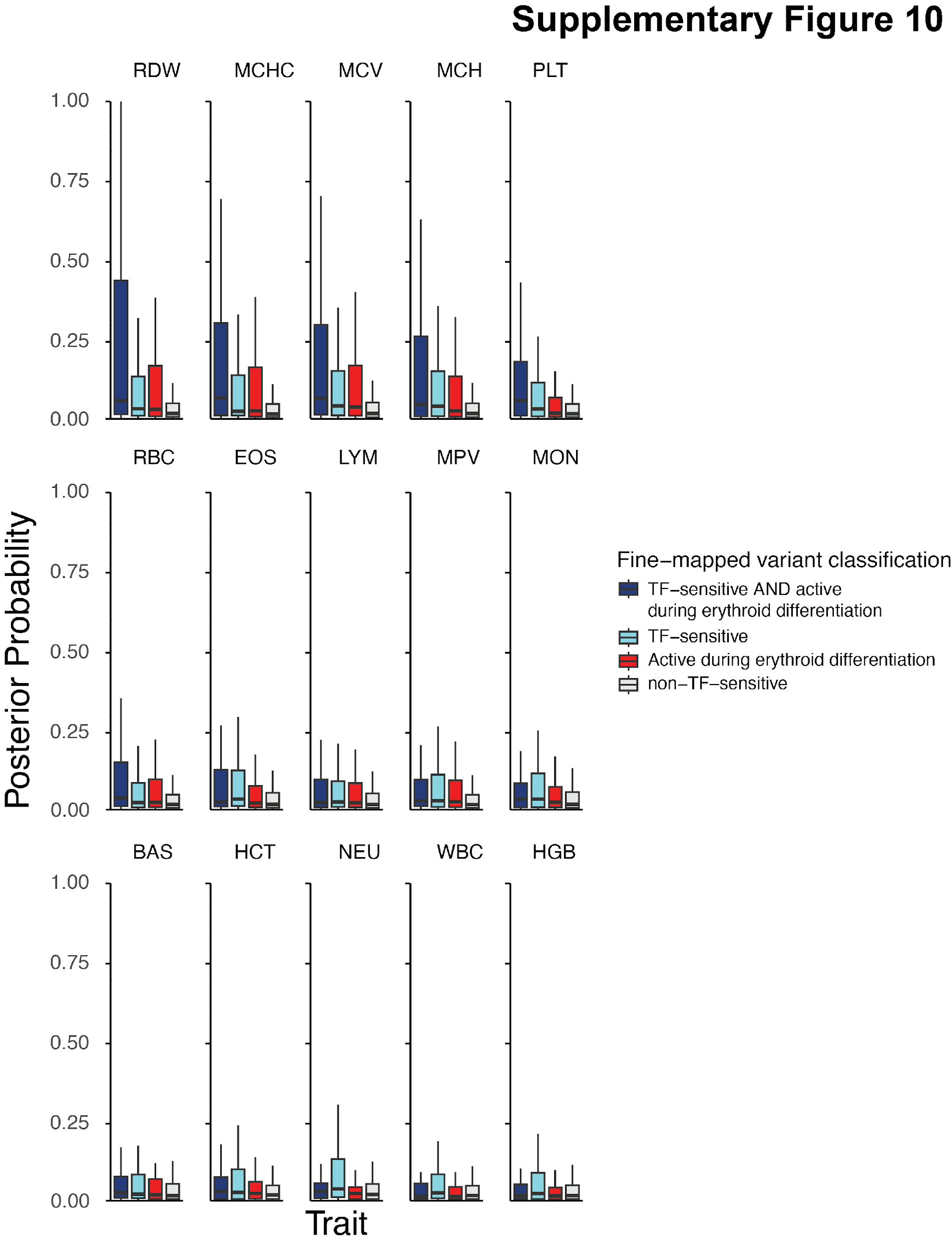


**Supplementary Figure 10.** **Posterior probability distributions for fine-mapped variants for blood phenotypes overlapping with ACRs of interest in our study.** Boxplots of the posterior probability for variants with posterior probability >0.01 overlapping each category, colored by category. Blood cell traits are referred as follows in the figure: BAS = basophil count, EOS = eosinophil count, HCT = hematocrit, HGB = hemoglobin, LYM = lymphocyte count, MCH = mean corpuscular hemoglobin, MCHC = mean corpuscular hemoglobin concentration, MCV = mean corpuscular volume, MON = monocyte count, NEU = neutrophil count, PLT = platelet count, RBC = red blood cell count, RDW = red cell distribution width, WBC = white blood cell count. BAS, EOS, MON, NEU, WBC are non-erythroid myeloid traits; LYM is a lymphoid trait; PLT is a megakaryocytic trait; HCT, HGB, MCH, MCHC, MCV, RBC, RDW are erythroid traits.

**
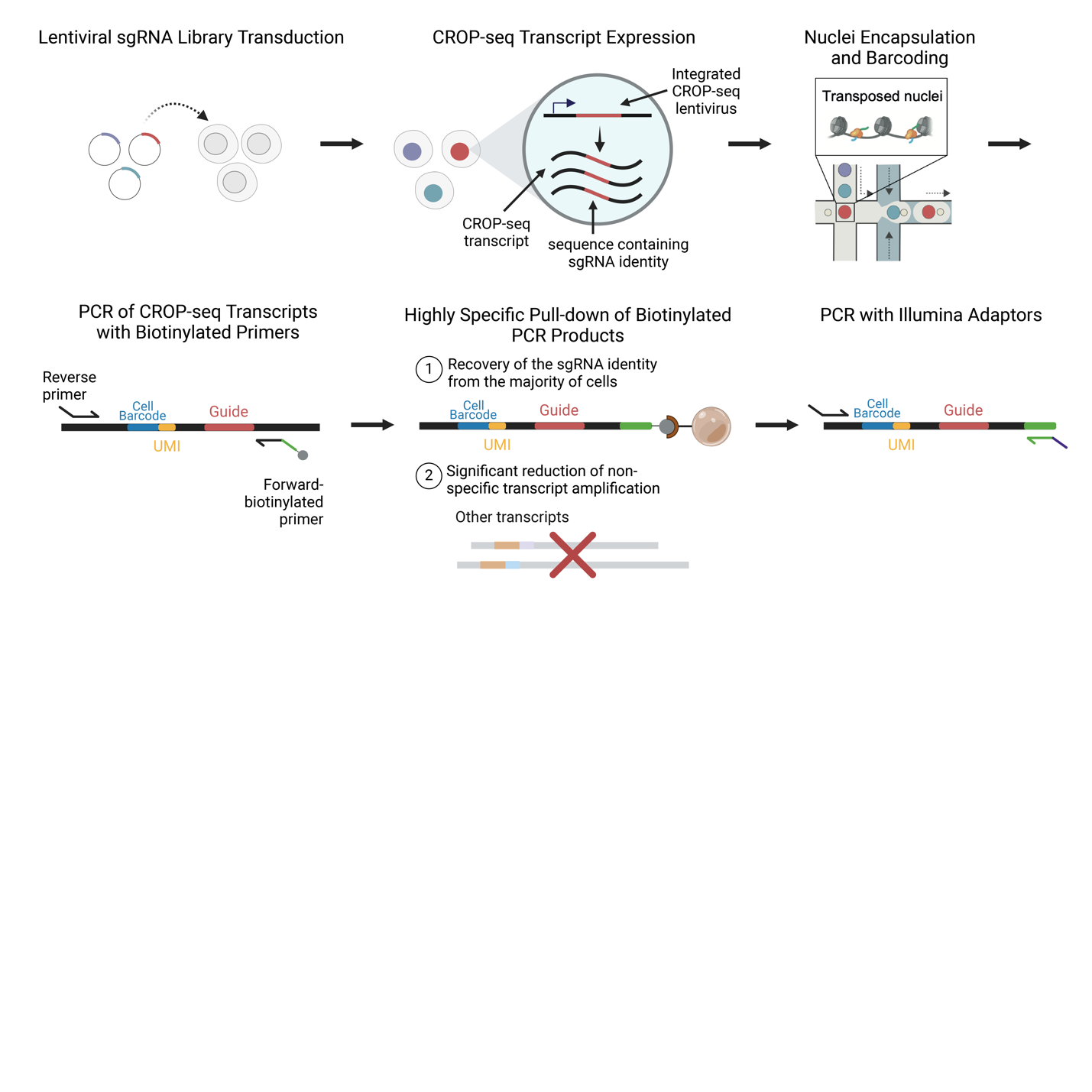
**

**Supplementary Figure 11. Schematic of Perturb-multiome’s biotinylation enrichment procedure to retrieve the identity of the sgRNA.** Created in BioRender. Lee, S. (2025) [https://BioRender.com/u14e945](https://biorender.com/u14e945).


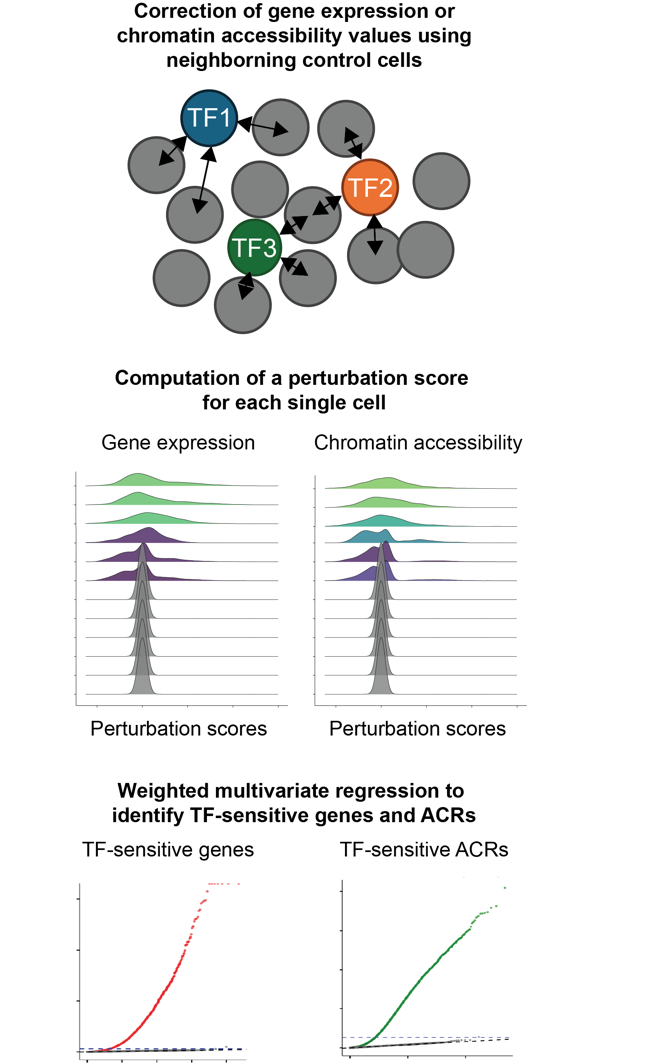


**Supplementary Figure 12.** Simplified schematic representation of the steps of the Mixscale framework. Full details can be obtained in the Mixscale preprint [*(*](https://paperpile.com/c/OfZ7hU/7lTLN)*27*[*)*](https://paperpile.com/c/OfZ7hU/7lTLN). Schematic steps throughout Mixscale identification of perturbations. (a) Each cell assigned with a perturbation is compared to the nearest control (NT) neighbors for (b) RNA or ATAC dimension to calculate a continuous perturbation score per each cell. (c) The perturbation score is leveraged to weigh a multivariate regression to identify TF-sensitive genes or ACRs, so cells with high perturbation scores contribute more to the regression.


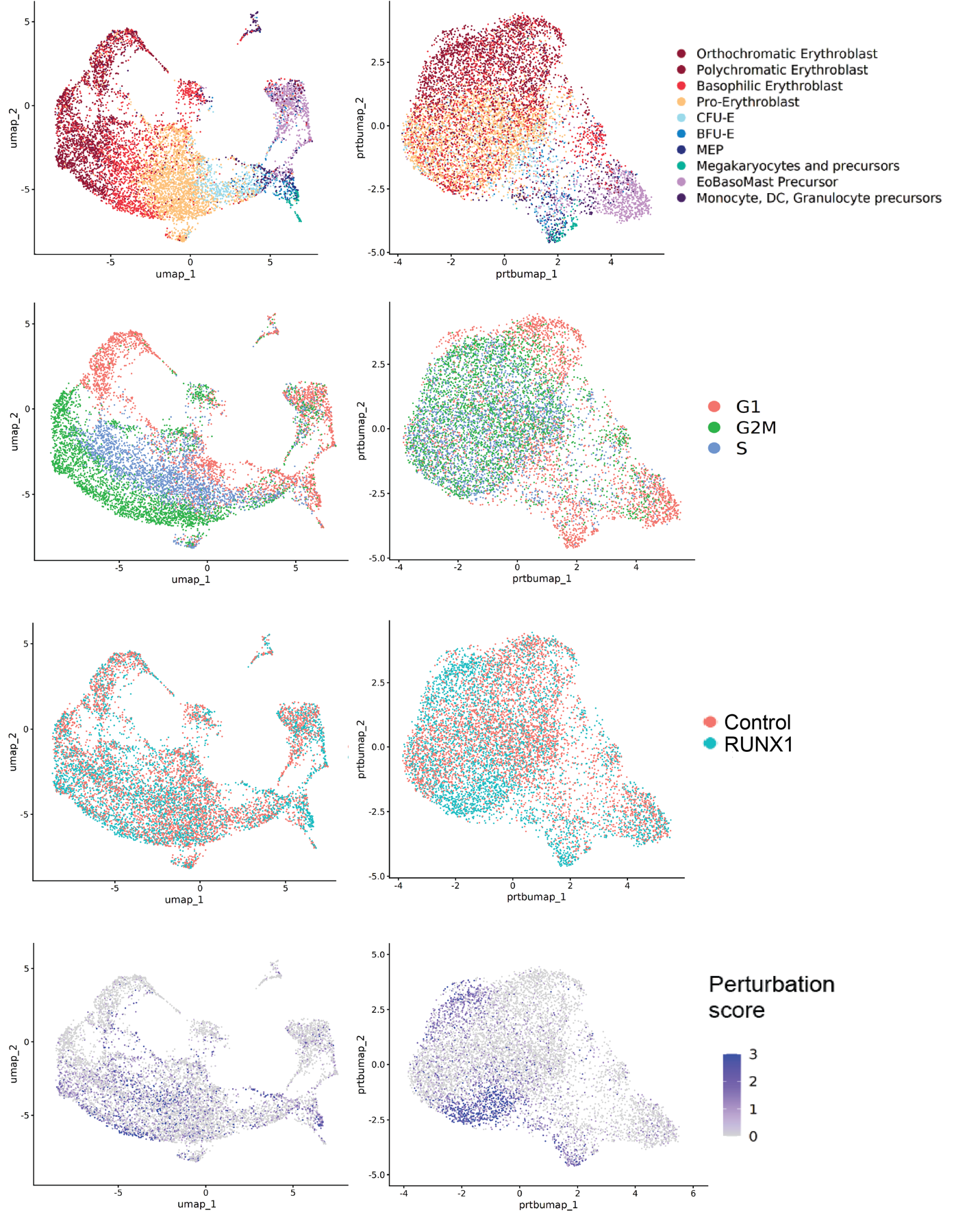


**Supplementary Figure 13. Calculation of local perturbation signatures.** Left, UMAP on the scRNA-seq space. Right, UMAP based on local perturbation signatures. Rows color cells by cell type, cell cycle stage, sgRNA identity and perturbation score, respectively, for equal numbers of cells with control sgRNAs and RUNX1-targeting sgRNAs. Local perturbation signatures were calculated using all cells in the experiment.


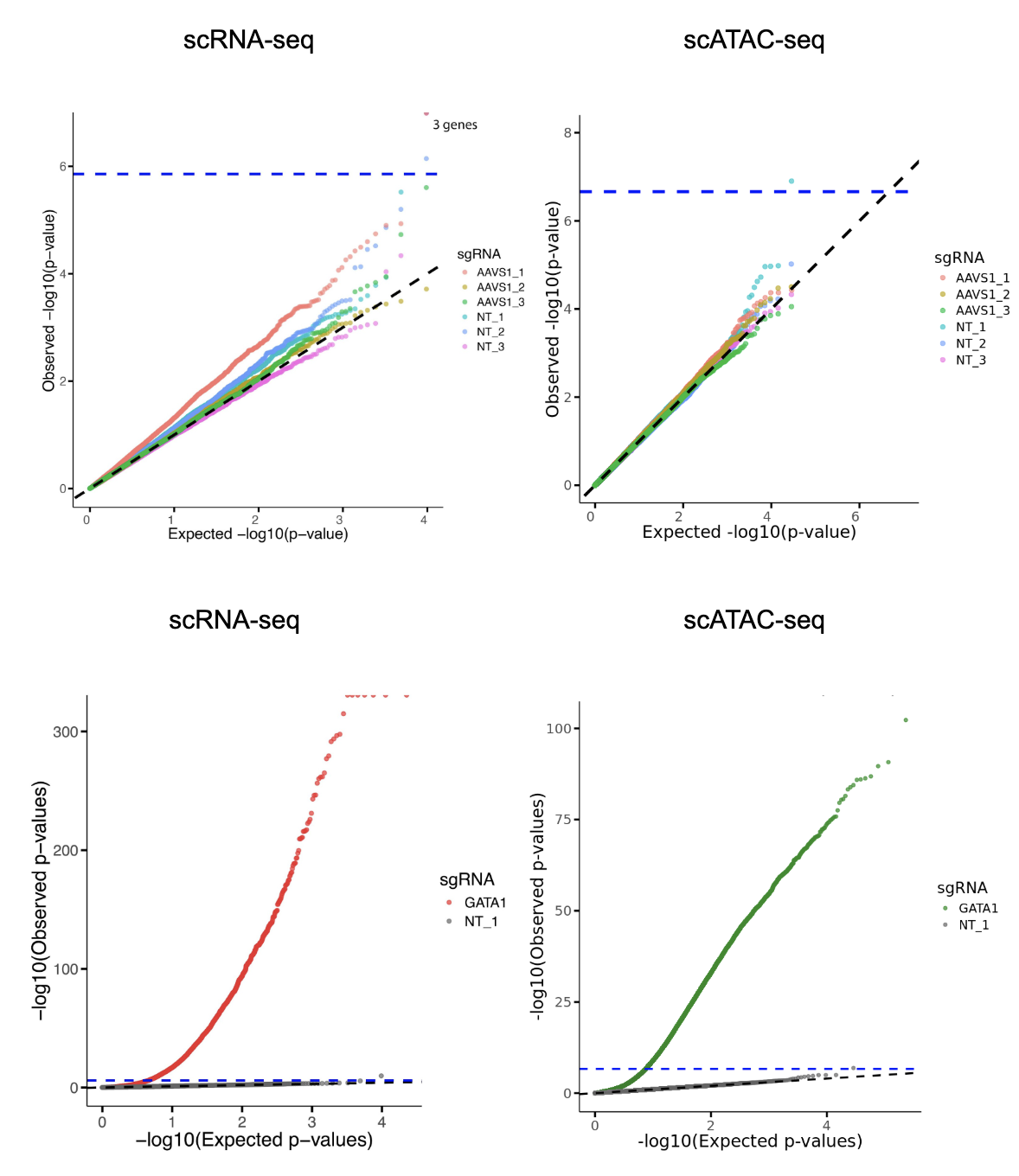


**Supplementary Figure 14.** Calibration of the discovery of TF-sensitive genes and ACRs. Top left, distribution of observed versus expected -log_10_(p-values) of differentially expressed genes obtained by comparing each control sgRNA to a combination of the 5 others. Top right, Distribution of observed versus expected -log_10_(p-values) of differentially accessible ACRs obtained by comparing each control dataset to a combination of the 5 others. Bottom left, distribution of observed versus expected -log_10_(p-values) of differentially expressed genes identified in cells with GATA1 sgRNAs or with one of the control sgRNAs. Bottom right, distribution of observed versus expected -log_10_(p-values) of differentially expressed genes identified in cells with GATA1 sgRNAs or with one of the control sgRNAs. In all plots, the black dashed line represents the observed = expected p-values, and the blue dashed line represents the p-value threshold adjusted for multiple comparisons, as used for the discovery of TF-sensitive genes.

**Table S1.**

Sequence of sgRNAs cloned in the CROP-seq vector and position of the cut site in the coding sequence of each target gene.

**Table S2.**

List of Transcription Factor-sensitive Accessible Chromatin Regions per perturbation.

**Table S3.**

List of TF-sensitive genes per perturbation.

**Table S4.**

Multiparametric Flow Cytometry Antibodies.

**Table S5.**

Sequence of oligonucleotides used in single cell genotyping protocol, readout of sgRNA identity, PCR and RT-qPCR.

**Table S6.**

LDSC results.
